# Empowering Adsorption and Photocatalytic Degradation of Ciprofloxacin on BiOI Composites: A Material-by-Design Investigation

**DOI:** 10.1021/acsomega.3c06243

**Published:** 2023-11-06

**Authors:** Sepideh G. Khasevani, Dariush Nikjoo, Cécile Chaxel, Kentaro Umeki, Shokat Sarmad, Jyri-Pekka Mikkola, Isabella Concina

**Affiliations:** † Department of Engineering Sciences and Mathematics, Luleå University of Technology, 98187 Luleå, Sweden; ‡ Wallenberg Wood Science Center, Department of Chemistry Technical Chemistry, Department of Chemistry, Chemical-Biological Centre, Umeå University, SE-90871 Umeå, Sweden; § Industrial Chemistry & Reaction Engineering, Johan Gadolin Process Chemistry Centre, Åbo Akademi University, FI-20500 Åbo-Turku, Finland

## Abstract

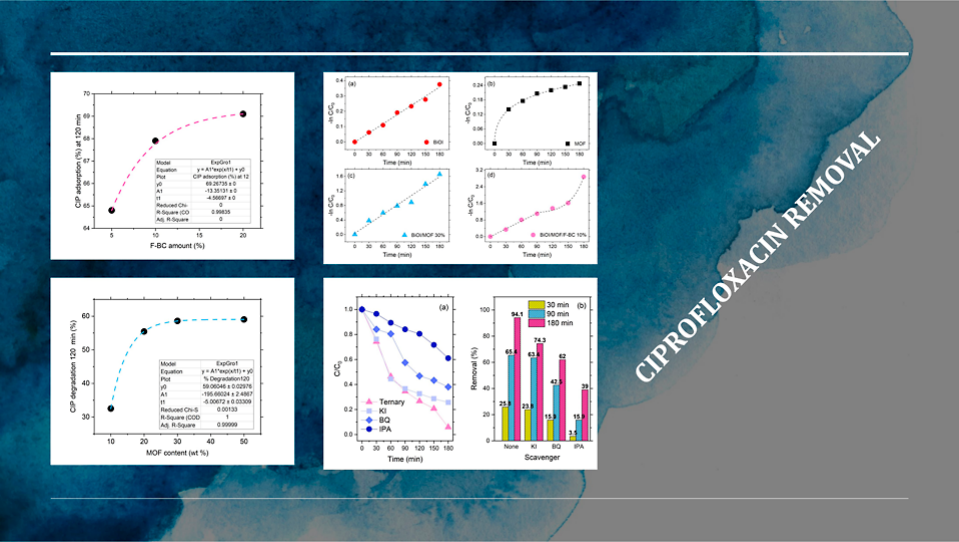

Binary and ternary composites of BiOI with NH_2_-MIL-101(Fe) and a functionalized biochar were synthesized through an *in situ* approach, aimed at spurring the activity of the semiconductor as a photocatalyst for the removal of ciprofloxacin (CIP) from water. Experimental outcomes showed a drastic enhancement of the adsorption and the equilibrium (which increased from 39.31 mg g^–1^ of bare BiOI to 76.39 mg g^–1^ of the best ternary composite in 2 h time), while the kinetics of the process was not significantly changed. The photocatalytic performance was also significantly enhanced, and the complete removal of 10 ppm of CIP in 3 h reaction time was recorded under simulated solar light irradiation for the best catalyst of the investigated batch. Catalytic reactions supported by different materials obeyed different reaction orders, indicating the existence of different mechanisms. The use of scavengers for superoxide anion radicals, holes, and hydroxyl radicals showed that although all these species are involved in CIP photodegradation, the latter play the most crucial role, as also confirmed by carrying out the reaction at increasing pH conditions. A clear correlation between the reduction of BiOI crystallite sizes in the composites, as compared to the bare material, and the material performance as both adsorbers and photocatalyst was identified.

## Introduction

The unwanted release of antibiotic residuals in waters has over time caused the insurgence of antimicrobial resistance, fighting which has been recognized as a priority for global public health. In 2015, WHO issued a global action plan on antimicrobial resistance and urged each country to formulate its own plan.^1^ This problem has become even more acute due to the COVID-19 pandemic: according to a recent evaluation, 75% of patients affected by Sars-CoV-2 were given antibiotics,^2^ and the extent of the associated water pollution is still hard to estimate. The capability to clean water systems has since then become even more urgent than it used to be.

Different technologies are proposed to face this huge challenge using both physical (isolation and removal of the pollutants) and chemical strategies (transformation of the pollutants into less harmful substances).^3^ However, no single approach is capable of facing the water pollution issue as a whole, and a multitechnique strategy should be considered.

Within this framework, engineering efficient, durable, robust, and easy-to-recover adsorbents and photocatalysts can play an important role in contributing to water remediation by either the adsorption of the organic target pollutant or its conversion to less harmful compounds (or in the best case its complete oxidation to CO_2_ and water) or by a combination of these two processes.

Among the bismuth oxyhalides, BiOI is attracting particular attention as a photocatalyst, thanks to its layered structure,^4,5^ which is supposedly efficient in transferring photogenerated electron–hole pairs, thus promoting the redox reactions subsequently responsible for the degradation of the target pollutants. Furthermore, the small band gap (around 1.8 eV) of BiOI renders it suitable for visible light absorption.

Metal–organic frameworks (MOFs) have recently emerged as potent candidates for photocatalytic water remediation, thanks to their porous structure, high specific surface area, and modifiable structure and properties.^6−9^ Among them, iron-based MOFs showed excellent results in Fenton catalysis due to the easy charge exchange in the Fe^II^/Fe^III^ couple, which, however, needs a partner to support its recycling. In this sense, it has been recently proven that the –NH_2_ functionalization of MIL-101(Fe) is a winning strategy to promote the Fe^III^ → Fe^II^ half-reaction kinetics, thus continuously generating hydroxyl radicals.^10^

Biochar, and more in general porous carbon-based materials, is gaining fame well as an adsorbent/catalytic material, alone or in combination with other catalytic systems, owing to its peculiar characteristics such as high porosity, high surface area, stability, recoverability, and possibility of insertion of functional groups.^11−15^ Recent literature shows that integrating biochar in transition metal compounds is very beneficial to spurring the catalytic performance of the native materials. For instance, the *in situ* growth of NiCo_2_O_4_ nanosheets on waste biomass-derived carbon resulted in enhanced performance in the removal of phenolic compounds, attributed to the increased exposure of more M–O–M induced by the carbon material and to its intrinsic high specific surface area.^16^ Biochar was also proved useful in supporting iron disulfide, grown on its surface, to activate peroxymonosulfate in the removal of tetracyclines and dyes by reducing the Fe leaching as compared to bare FeS_2_.^17^

The fabrication of hybrid catalysts, consisting of materials featuring complementary skills in terms of adsorption and catalytic activity, in the attempt to create a synergistic effect, is considered a powerful strategy to introduce robust and reliable systems for water remediation.^18−22^

In the present investigation, we engineered BiOI, with a metal–organic framework (MOF), namely, NH_2_-MIL-101(Fe), and with a COOH-functionalized biochar, aiming at empowering their performance as both adsorbents and photocatalysts to remove ciprofloxacin (CIP) from water.

## Results and Discussion

### Adsorption of CIP under Dark

**Figure 1:**
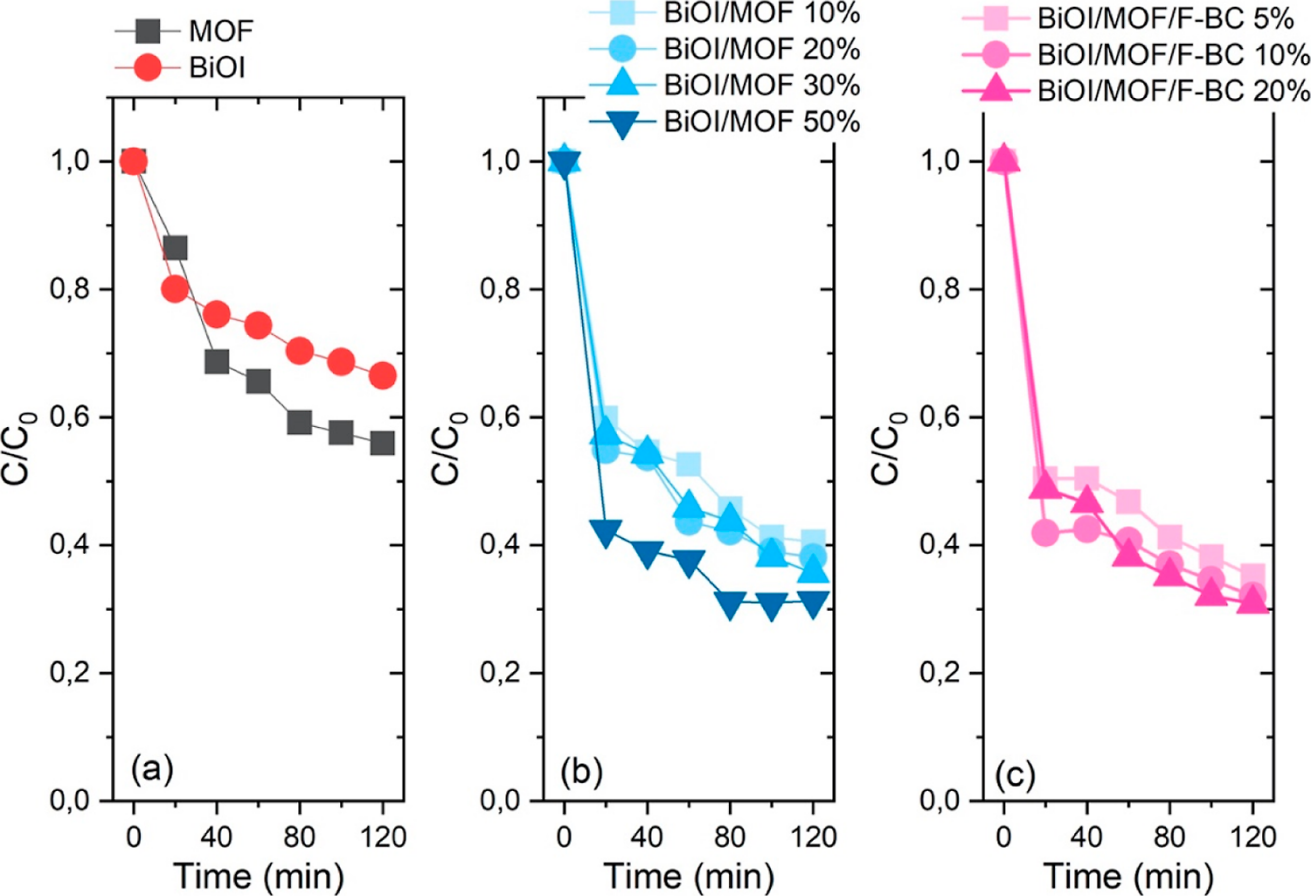
CIP adsorption plotted as *C*/*C*_0_ for the materials under investigation. Markers are experimental points, and lines are guides for the eye. (a) Bare materials; (b) binary composites; (c) ternary composites.

The adsorption skills of bare materials (BiOI and MOF), binary composites (BiOI/MOF-10,20,30,50%), and ternary composites with F-BC (BiOI/MOF/F-BC-5,10,20%) were investigated under dark conditions. The course of the reaction, displayed as the relative disappearance of CIP, *C*/*C*_0_, is reported in Figure 1.

Bare materials showed a moderate adsorption capability in the dark, as depicted in Figure 1 a (about 30 and 40% for BiOI and MOF, respectively, in 2 h). It should be noted that the adsorption–desorption equilibrium could not be reached after 2 h of reaction time.

The adsorption capacity of BiOI was drastically improved by the combination with NH_2_-MIL-101(Fe) as a hybrid material (Figure 1b). All the binary composites showed, indeed, an excellent capability of adsorbing CIP molecules, especially the sample BiOI/MOF-50%, which could uptake 70% of the initial CIP concentration in 70 min, with 60% of it removed from the solution during the first 20 min. This makes it an excellent candidate to be used as an adsorbent for CIP residuals in water. The other three binary composites (featuring 10, 20, and 30% MOF) showed similar performance (slightly more than 60% of the initial CIP concentration was adsorbed in 120 min).

All of the ternary composites gave rise to a similar absorption capability over the long run (Figure 1c). However, the small differences observed among these samples are significant from a material composition standpoint; a plot of the amount of CIP absorbed after 120 min under dark conditions *vs* the amount of F-BC in the composites demonstrated an excellent exponential trend (Figure S1 in the Supporting Information). Similar to what was observed in the case of the binary composites, the ternary materials also display an excellent and fast CIP absorption capability (50% for the samples containing F-BC as high as 5 and 20% and about 60% for the BiOI/MOF/F-BC 10% sample in 20 min), rendering them excellent candidates as adsorbents for CIP removal from water.

**Figure 2:**
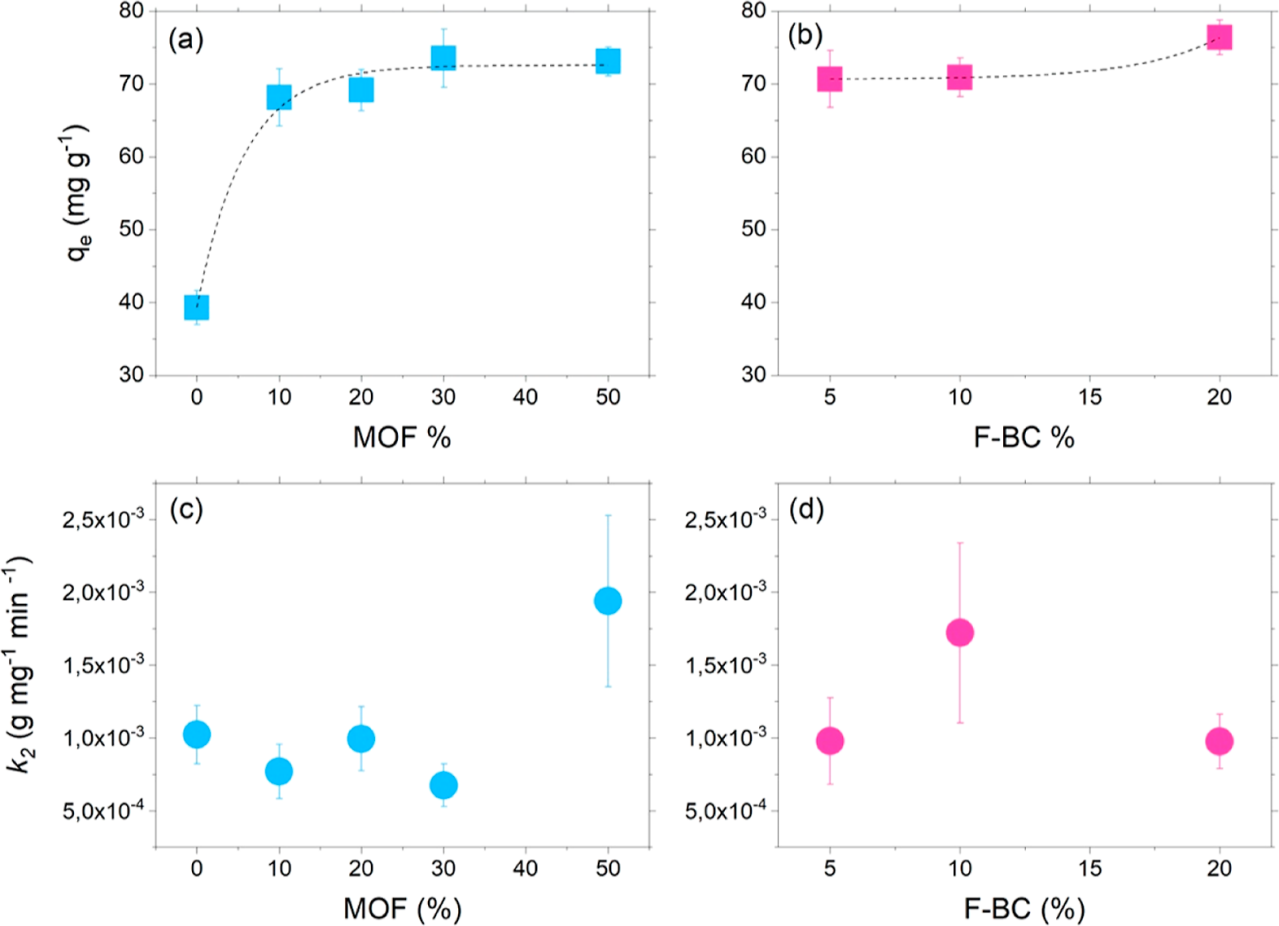
(a,b) Adsorption at the equilibrium *q*_e_ and (c,d) observed kinetic constants for the adsorption process for the materials under investigation as a function of the content of MOF and F-BC.

We quantified the adsorption capacity at the equilibrium using a pseudo-second-order model (to which all the materials resulted to obey)
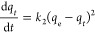
1whose integrated equation is
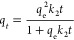
2where *q*_*t*_ (mg adsorbate/g adsorbent) is the absorption at a given time,^23^ according to
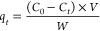
3and *q*_e_ is the adsorption at the equilibrium; *C*_0_ (mg L^–1^) is the initial concentration of CIP, *C*_*t*_ (mg L^–1^) is the concentration of CIP at a given time, *V* (L) is the solvent volume, and *W* (g) is the mass of adsorbent used in the adsorption process. The model fitting results and pertaining parameters are reported in the Supporting Information (Figure S2 and Table S1), while Table 1 reports the obtained values for *q*_e_ and the observed kinetic constants for the adsorption process, which are also plotted as a function of the amount of MOF and F-BC in Figure 2.

**Table 1:** Adsorption at the Equilibrium and Observed Adsorption Kinetic Constant for the Materials under Investigation, as Retrieved from the Pseudo-Second-Order Model Applied to the Experimental Outcomes

material	*q*_e_ (mg g^–1^)	*k*_2_ × 10^–3^ (g mg^–1^ min^–1^)
BiOI	39.31 ± 2.35	1.022 ± 0.200
BiOI/MOF 10%	68.17 ± 3.93	0.7698 ± 0.186
BiOI/MOF 20%	69.16 ± 2.83	0.9936 ± 0.221
BiOI/MOF 30%	73.53 ± 4.02	0.6739 ± 0.147
BiOI/MOF 50%	73.10 ± 2.01	1.938 ± 0.589
BiOI/MOF/F-BC 5%	70.67 ± 3.91	0.9787 ± 0.297
BiOI/MOF/F-BC 10%	70.92 ± 2.67	1.721 ± 0.619
BiOI/MOF/F-BC 20%	76.39 ± 2.38	0.9751 ± 0.186

The adsorption at equilibrium *q*_e_ was found to be an exponential function of the material’s composition, for both the binary and ternary composites (Figure 2a,b). The BiOI/MOF adsorbents showed a plateau in the value of *q*_e_ for a MOF amount as high as 30%, *i.e.*, further addition of MOF did not spur further the uptake of the CIP molecules (Figure 2a). On the other hand, a significant increase in *q*_e_ for the ternary composites (Figure 2b) was only found for the highest amount of F-BC inserted in the material (20%).

The observed kinetic constants for the adsorption process (plotted as a function of material composition in Figure 2c,d) did not give rise to any significant differences among the investigated materials; the kinetics of the process are not influenced by the material composition.

The observed findings for the adsorption process are summarized as follows: (i) the coupling of BiOI with the MOF significantly impacted the adsorption capability of the materials, almost doubling the adsorption at the equilibrium of BiOI when 30% MOF is added (); (ii) further insertion of functionalized biochar to the binary composite did not cause a significant increase in the value of *q*_e_; and (iii) the kinetics of the process is not relevantly affected upon changing the material composition.

### Photocatalytic Performance

**Figure 3:**
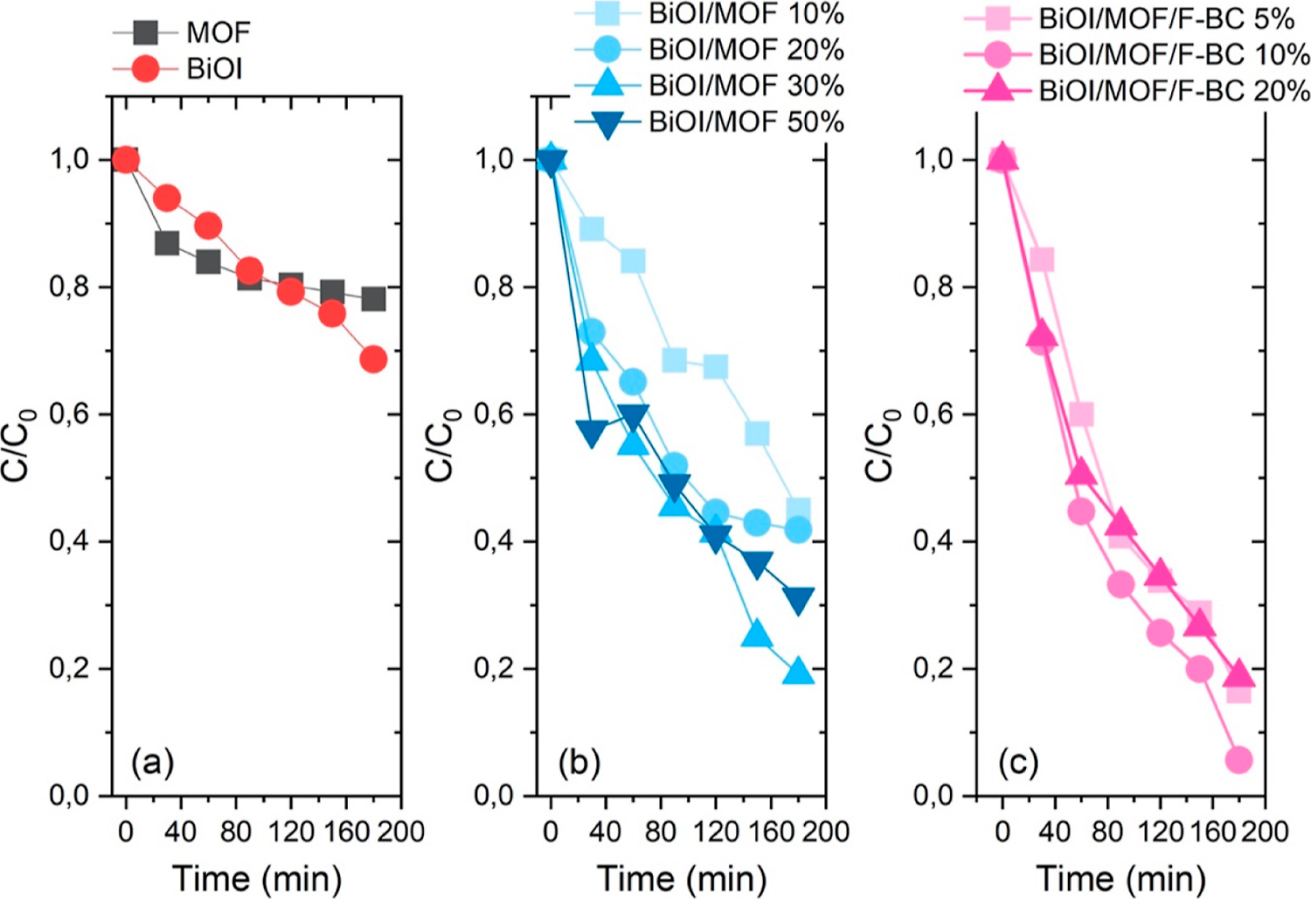
Reaction course for the CIP degradation under simulated solar light for the investigated materials: (a) bare materials; (b) binary composites; (c) ternary composites. Markers are experimental points, and lines are guide for the eye.

The catalytic activity of bare materials (BiOI and MOF), binary composites (BiOI/MOF-10,20,30,50%), and ternary composites with F-BC was investigated under simulated solar light (the reaction courses are shown in Figure 3).

The catalytic activity of BiOI and MOF upon simulated solar light (Figure 3a) was rather poor, with BiOI overperforming the MOF by 10% in 3 h. Overall, only 30% of the initial CIP concentration was removed in a rather long reaction time (180 min).

On the contrary, the binary compounds showed a good capability of degrading CIP (Figure 3b); the addition of MOF to BiOI resulted in enhanced catalytic performance, which correlated very well with the percentage of MOF in the composites according to an exponential trend up to 120 min reaction time (see Figure S3 in the Supporting Information), after which the binary composites featuring 30% MOF overperformed all the homologues.

We monitored the reaction for 180 min, verifying the following: (i) the insertion of 10% MOF results in a fair CIP degradation at long reaction times, but the reaction kinetics is the slowest of the investigated batch; (ii) at long reaction times, the composites featuring 10 and 20% MOF did not significantly differ in terms of their CIP degradation capability; (iii) the composites with 30 and 50% MOF content display similar catalytic performance, but over the long run, the former is capable of removing more antibiotics (it was then chosen as the base material to synthesize the ternary composites).

Upon simulated solar light irradiation, all of the ternary composites showed an excellent capability of degrading CIP (Figure 3c): after 1 h, between 40 and 60% of the initial CIP amount was removed. At longer reaction times (180 min), we observed the disappearance of almost 95% when the process was catalyzed by the sample containing 10% of F-BC (the best of the batch, whose recyclability was tested as well; see Figure S4 in the Supporting Information), while the other two catalysts could remove between 75% (BiOI/MOF/F-BC 20%) and 83% (BiOI/MOF/F-BC 5%).

Catalytic results are summarized in Table 2.

**Table 2:** Summary of the Photocatalytic Results

material	CIP degradation under light @ 180 min (%)
BiOI	31.3
MOF	21.9
BiOI/MOF 10%	55.0
BiOI/MOF 20%	58.2
BiOI/MOF 30%	81.9
BiOI/MOF 50%	68.8
BiOI/MOF/F-BC 5%	83.5
BiOI/MOF/F-BC 10%	94.4
BiOI/MOF/F-BC 20%	75.6

**Figure 4:**
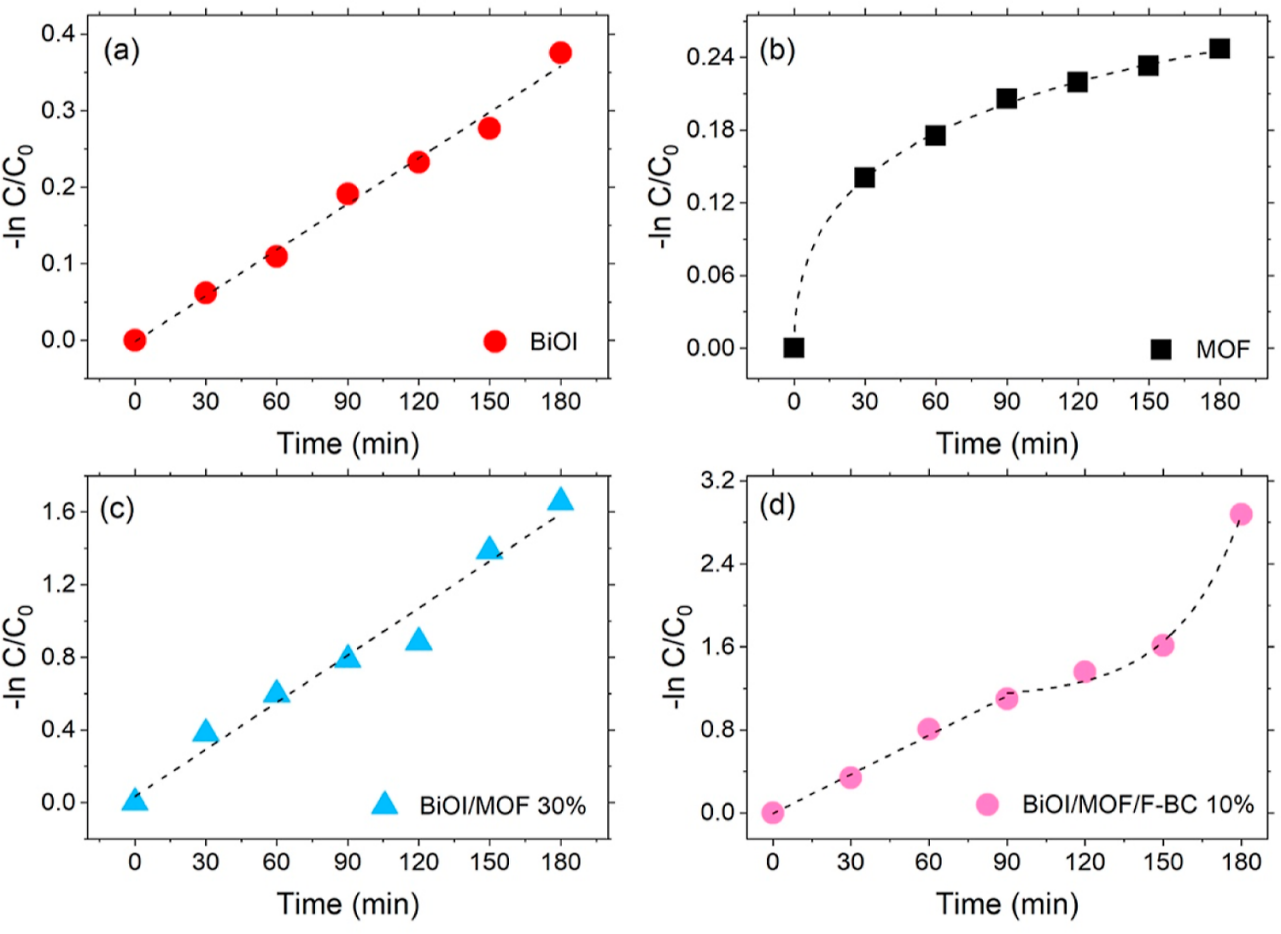
Reaction course for the degradation of CIP upon light irradiation plotted as −ln(*C*/*C*_0_) for pure BiOI (a), BiOI (b), BiOI/MOF-30% (c), and BiOI/MOF/F-BC-10% (d). Markers are experimental points, and lines are fitting curves.

A deeper analysis of the reaction courses for the bare materials (BiOI and MOF) and the best-performing composites (BiOI/MOF 30% and BiOI/MOF/F-BC 10%) allowed us to observe that different catalytic materials obey different reaction mechanisms (Figure 4 and Table S2 in the Supporting Information).

Specifically, we observed a well-behaved linear behavior in the case of CIP degradation degraded by pure BiOI (Figure 4a) which, however, appears to be associated with a pseudo zero-order reaction, since *C*/*C*_0_*vs* time is also linear (see Figure S5 in the Supporting Information).

A reaction order higher than one was instead found for the reaction catalyzed with bare MOF (Figure 4b), which followed a sigmoidal trend, highlighting the occurrence of an autocatalytic reaction. This indicates that NH_2_-MIL-101(Fe) is an active catalyst, although sloppy, for the degradation of CIP also under dark conditions. This was expected from previous literature, where the material was used to catalyze the degradation of several molecular targets, through the activation of H_2_O_2_, without the need to be light-activated.^6,8,10^ The presence of reaction products in the reaction mixture when the light was switched ON promoted the reaction at early reaction times (up to 90 min), as is visible in Figure 3a, during which the MOF slightly outperformed bare BiOI.

The reaction supported by binary composite BiOI/MOF 30% displayed pseudo-first-order kinetics (Figure 4c). It is interesting to remark that the absence of a sigmoidal trend indicates that MOF is no longer active as a catalyst under dark when combined with BiOI, suggesting that the interaction between the Fe(III)/Fe(II) species in the MOF and the BiOI is favored, as compared with the interaction with the MOF with the surrounding environment (*i.e.*, the CIP molecules in the reaction mixture). Furthermore, the catalytic behavior of the binary composite is changed, as compared with that of the bare materials, resulting in a different reaction order, suggesting a different reaction mechanism, induced by the different nature of the catalytic material.

When the ternary composite BiOI/MOF/F-BC 10% was used as the catalyst, a more complex behavior was identified (Figure 4d). Indeed, the semilog plot of the reaction course under light irradiation showed two different regimes: a linear trend (pseudo-first-order kinetics) up to 90 min reaction, followed by an exponential (higher kinetic order) trend. This change is attributed to the presence of the functionalized biochar; the generation of acidic group resulted in the possibility of hydrogen-bonding CIP thus promoting its degradation.^12^

Fitting equations and *R*^2^ values are reported in Table S2 in the Supporting Information.

### Influence of pH Conditions on CIP Degradation

**Figure 5:**
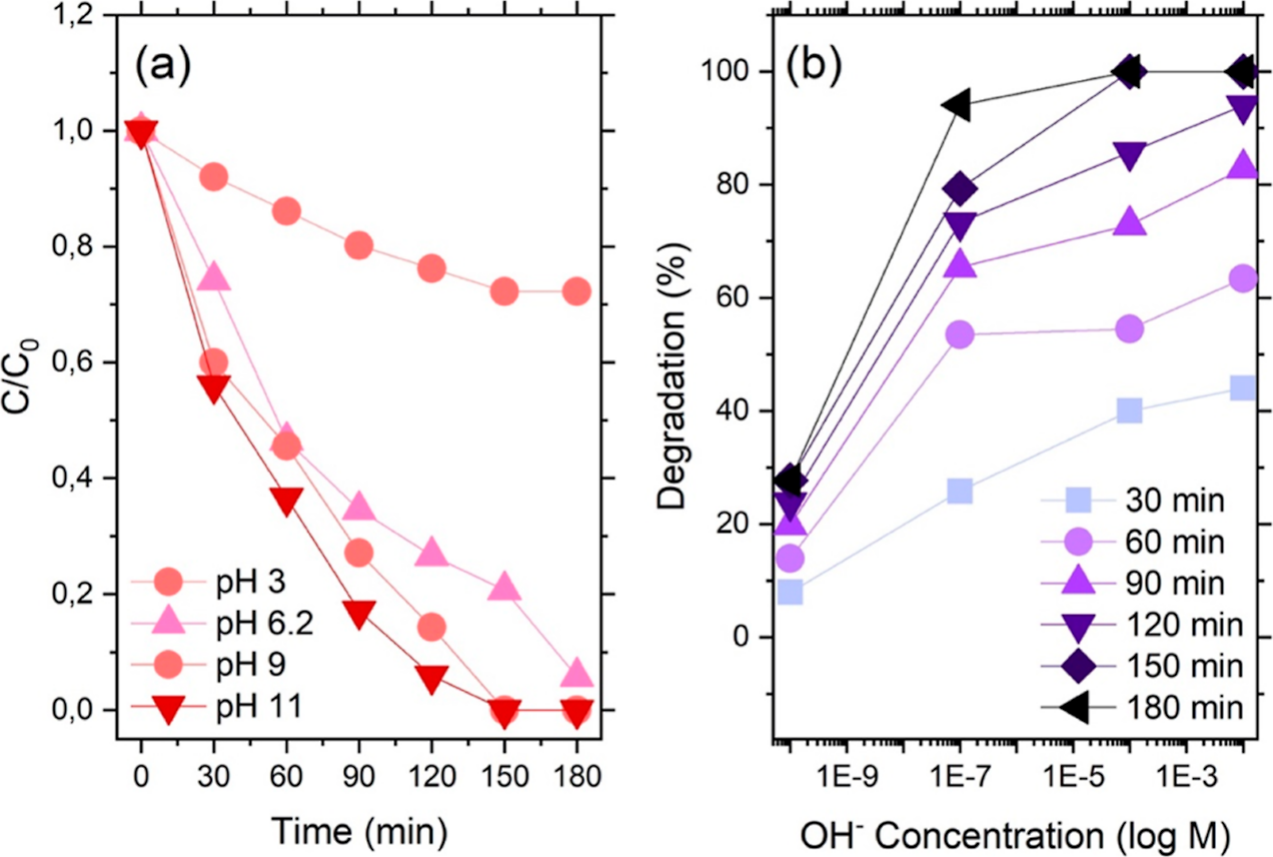
(a) Reaction course plotted as *C*/*C*_0_*vs* time for the degradation of CIP as a function of the reaction mixture pH (pH = 6.2 corresponds to the CIP autodissociation). (b) Percentage of degraded CIP *vs* OH^–^ concentration at different reaction times.

CIP degradation, photocatalyzed by the ternary composite BiOI/MOF/F-BC 10%, was studied under different acidic/alkaline conditions (the results are shown in Figure 5). Forcing a certain pH implies imposing a change in the CIP form, with its acidic dissociation constants equal to p*K*_a1(COOH)_ = 6.09 and p*K*_a2(NH)_ = 8.74.

It was previously reported that strong alkaline conditions favor CIP degradation, possibly due to an easier UV excitation of the antibiotics.^24^ We observed the same in the present study (Figure 5a); in an acidic pH, with CIP fully protonated, the reaction was slow and poorly efficient, and less than 30% of the initial CIP amount (10 ppm) was removed. Increasing the pH up to and beyond 9 (with both the carboxylic group and the NH on the piperazinyl ring deprotonated) resulted in a much faster and more efficient reaction (especially at initial times) and complete CIP removal after 150 min. The comparison of the outcomes for the reaction carried out at pH 9 and 11 highlighted small but significant differences in terms of the amount of degraded CIP at the same reaction time (Figure 5b). This finding indicates that not only the form of CIP is relevant to its removal but also the concentration of the hydroxyl ions in the solution (see the following section on the reaction carried out in the presence of ROS scavengers).

### Identification of the Reactive Oxidizing Species

**Figure 6:**
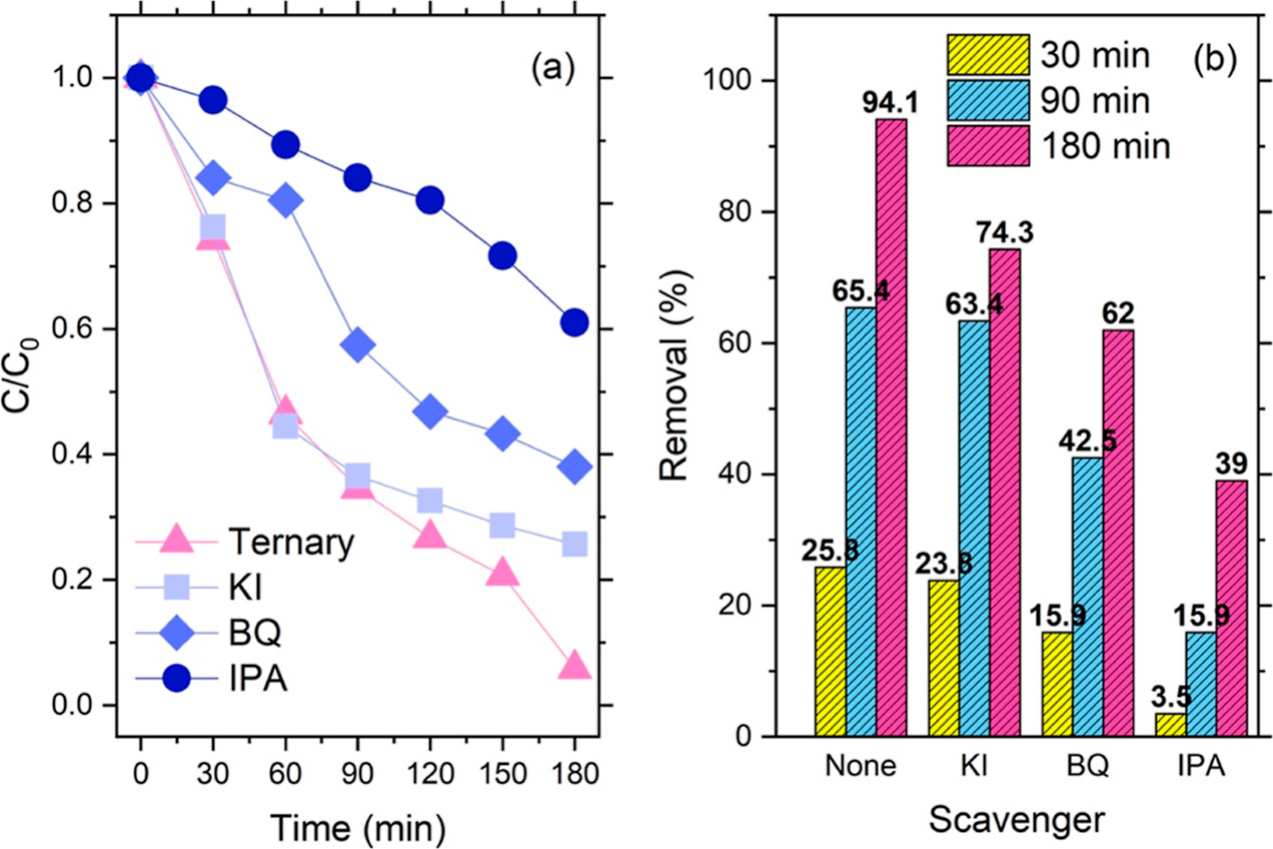
(a) Reaction course for the photodegradation of CIP under the action of different trapping agents. (b) Comparison of CIP removal at different reaction times for different trapping agents.

We finally investigated the photodegradation of CIP in the presence of different scavenging agents, specifically potassium iodide (trapping holes), *p*-benzoquinone (trapping the superoxide anion radical), and isopropyl alcohol (trapping the hydroxyl radical). The outcomes are reported in Figure 6. All trapped species seemed to have a role in the CIP degradation catalyzed under light irradiation by the ternary composite, again highlighting the complexity of this reaction. However, this role is played to different extent by different species. The analysis of the course of the reaction (Figure 6a) showed that the photogenerated holes are not the main species degrading CIP; their removal affects the course reaction only at prolonged reaction times (>90 min). Their recombination rate with the photogenerated electrons is possibly faster than the redox processes involved in CIP destruction. This was also confirmed by the fact that we could not observe any clear correlation between the absorption features of the materials and the amount of CIP degradation.

The removal of superoxide anion radical, on the other hand, affected more significantly the degradation of CIP over the time, and we observed only a 60% disappearance of the molecule in 3 h of reaction time.

Finally, we verified that the hydroxyl radicals played the most critical role in CIP removal. When IPA was added to the reaction mixture, the process slowed and significantly suppressed, indicating that these species dominate the redox reaction in charge of CIP degradation. A visual analysis of the relative role played by different trapping agents is provided in Figure 6b.

In an attempt to correlate the observed behaviors as adsorbents and photocatalysts with materials’ physical features, we analyzed the physical and chemical features of the bare materials (BiOI and the MOF) and the composites displaying the best functional performance (BiOI/MOF 30% and BiOI/MOF/F-BC 10%).

### Structural and Morphological Characterization

**Figure 7:**
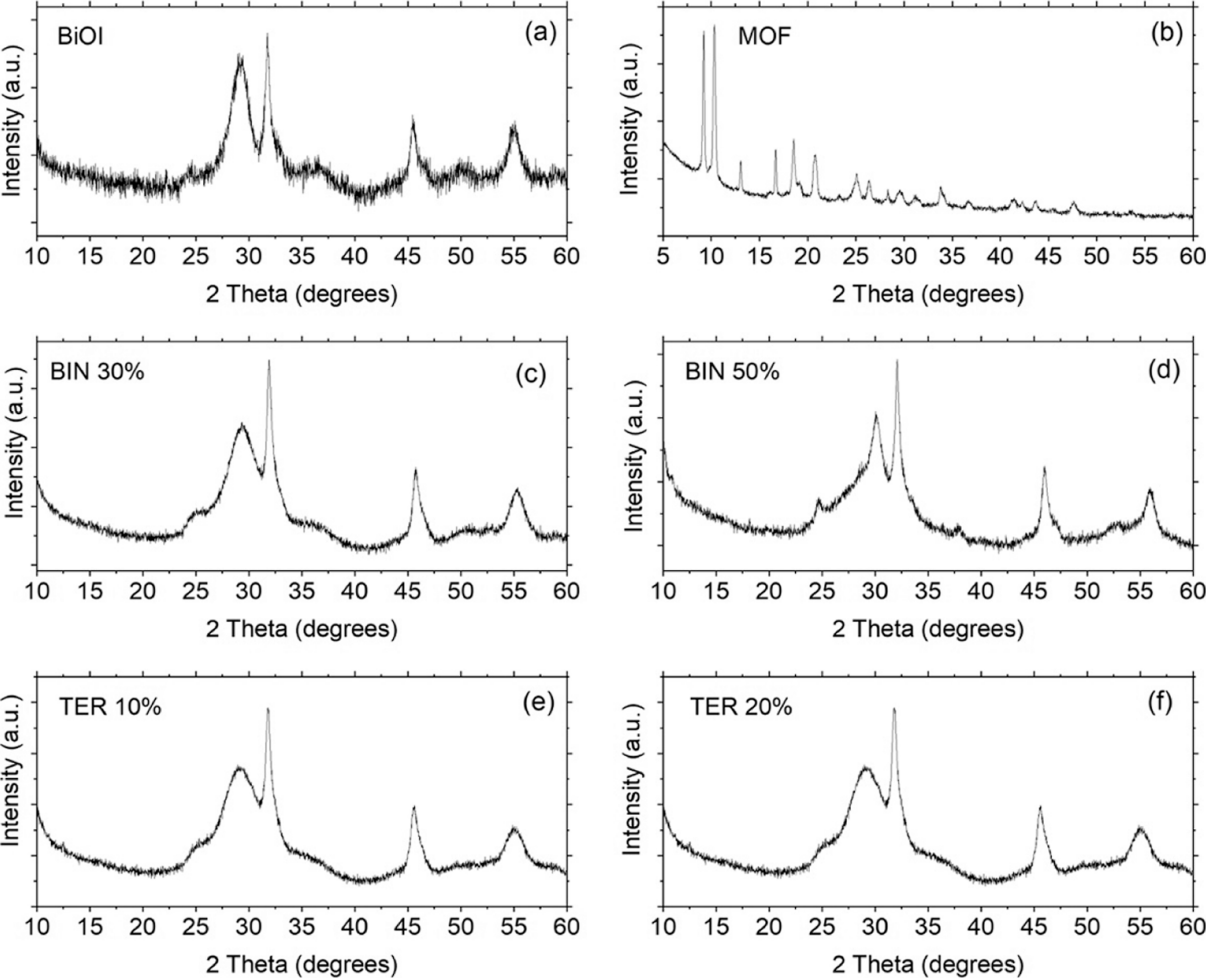
XRD patterns of the materials under investigation. (a) BiOI; (b) MOF; (c) BiOI/MOF 30%; (d) BiOI/MOF 50%; (e) BiOI/MOF/F-BC 10%; and (f): BiOI/MOF/F-BC 20%.

X-ray diffraction (XRD) patterns of the materials under investigation are reported in Figure 7. We focused our analysis on the possible changes induced in the BiOI structure during the *in situ* fabrication of the binary and ternary composites. Bare BiOI showed the reflections of planes (101), (102), (110), (200), (212) at 24.32, 29.2, 31.76, 45.53, and 54.85°, respectively (JCPDS card no. 00-73-2062), displaying the expected tetragonal structure. The same reflections are found in the XRD patterns of the composites, although slightly changed in terms of width and intensity, indicating that the fabrication process modified the native structure of BiOI. Reflections pertaining to the MOF are not visible in the analyzed composites (with the exception of a peak centered at 52.74° in the binary composite with the highest amount of MOF, *i.e.* 50%), indicating that BiOI structurally dominates the diffraction patterns. This was previously observed in the literature^7,25^ and can be ascribed to a high degree of dispersion of the MOF.

The XRD pattern of NH_2_-MIL-101(Fe) was in agreement with the literature.^26,27^ XRD analysis of the oxidized biochar (reported in Figure S6 in the Supporting Information) revealed the presence of two peaks, centered at 22.7 and 41.9°, ascribed to the plane indexes (002) and (100), respectively. Both peaks are asymmetric, rather broad, and feature small intensity. The asymmetry of the (002) reflection is associated with the parallel packing of the carbon layers and indicates the presence of amorphous and aliphatic structures.^28^ The (100) reflection is due to the diffraction of hexagonal graphene carbons.^29^

The quantitative analysis of the structural parameters of BiOI considered bare and in the composites (reported in Tables 3 and S2 in the Supporting Information) showed the following: (i) the native structure of BiOI is reproduced in all the composite materials, with small differences in the unit cell sizes; (ii) the interplanar distance *d* is found unchanged by comparison between pure BiOI and BiOI in the composites; (iii) different amounts of MOF added to BiOI cause a reduction in its average crystallite size of about 17% (MOF 30%) and 6% (MOF 50%); (iv) the decrease in the average crystallite size is much higher when F-BC is inserted in the composites (55%), but different amounts of F-BC resulted in the same average crystallite size. This finding indicates that the amount of MOF (the same in both ternary composites) rules the formation of BiOI. Our hypothesis is that we are herein observing an electrostatic interaction between the BiOI precursor and the MOF [possibly between the negatively charged oxygen atoms in Bi(NO_3_)_3_ and the positively charged –NH_2_ groups in NH_2_-MIL-101(Fe)], which dictates the subsequent formation of BiOI.

**Table 3:** Structural Parameters (Unit Cell Sizes and Average Crystallite Size) Are Retrieved from XRD Analysis^a^

	unit cell sizes (Å)		
material	*a* and *b*	*c*	average crystallite size (nm)
BiOI	3.99	9.32	13.3
BIN 30%	3.97	9.40	11.0
BIN 50%	3.95	9.00	12.5
TER 10%	3.98	9.83	4.97
TER 20%	3.98	9.83	4.97

a The average crystallite size *D* was evaluated using Debye–Scherrer analysis: , where *K* is the shape factor (taken as 0.94), λ the X-ray wavelength (1.54178 Å), β the full width at half-maximum (fwhm)of the reflection used in the calculation, and θ the reflection angle.

**Figure 8:**
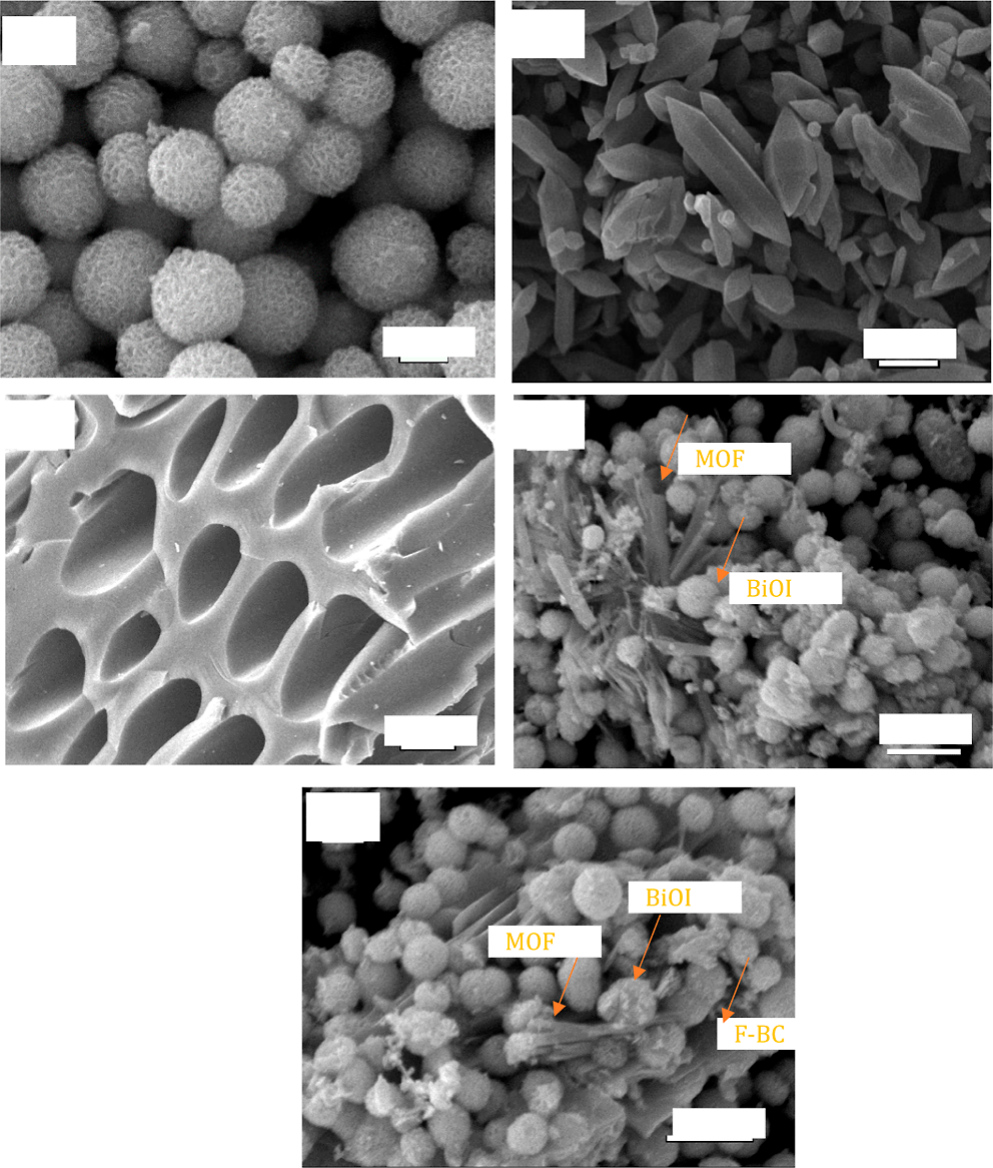
SEM images of the prepared materials. (a) BiOI, (b) MOF, (c) F-BC, (d) BiOI/MOF-30%, (e) BiOI/MOF/F-BC-10%.

Figure 8 reports the scanning electron microscopy (SEM) images of the samples under investigation. BiOI featured hierarchical 3D microspheres with an average diameter of 1.6 ± 0.2 μm (Figure 8a). As-synthesized MOF displayed a hexagonal microspindle shape, with length calculated around 1–3 μm (Figure 8b). The functionalized biochar showed a honeycomb-like morphology (Figure 8c), with pore sizes exceeding 5 μm. A porous structure is commonly reported for biochars,^30^ which should enhance the adsorption of organic molecules, thus spurring the subsequent catalytic degradation, thanks to a proximity effect.

Both the binary and ternary composites showed the incorporation of MOF as well as of F-BC in the BiOI structures (Figure 8d,e), which retained the same morphology they displayed when synthesized on their own.

### UV–Vis Absorption Spectroscopy

The interaction with light was studied by UV–vis absorption spectroscopy. This property is relevant in semiconductor-supported photocatalysts, and extension of light absorption should indeed enhance the photogeneration of charges useful for subsequent redox reactions, responsible for pollutant degradation.

**Figure 9:**
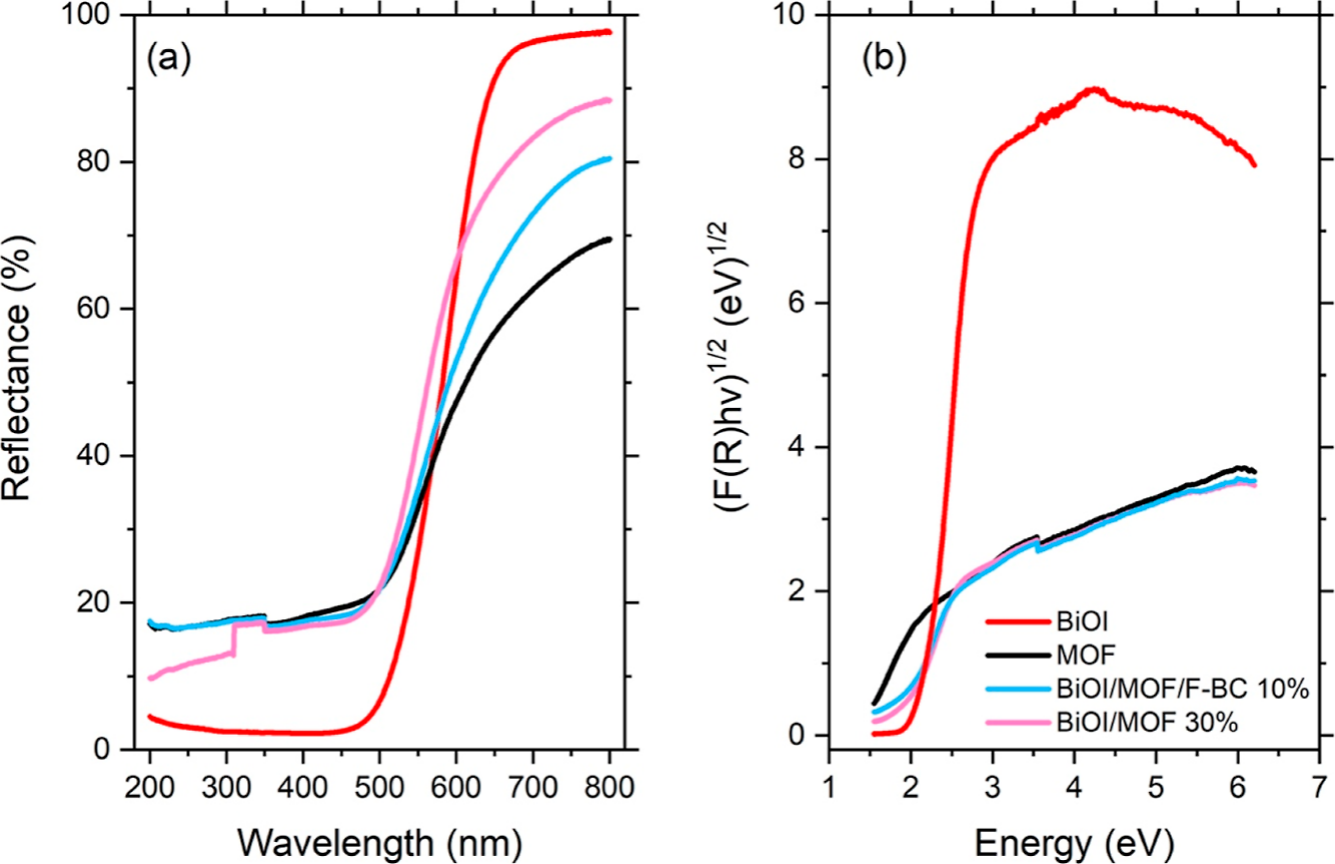
UV–vis absorption spectra (a) and Tauc plots (b) of the prepared materials.

The diffuse reflectance spectra of the prepared materials are shown in Figure 9a. All of the materials showed a high absorption up to around 500 nm, which then decreased to different amounts, depending on the specific compound. The addition of both MOF and F-BC extended light absorption to higher wavelengths as compared with that of bare BiOI.

The optical energy gap (*E*_g_) of the materials was calculated from the absorption data (Tauc plot shown in Figure 9b and values reported in Table 4). A significant reduction in the *E*_g_ was observed for the composite materials, compared to bare BiOI (1.84 eV); the optical energy gap was found to be as high as 1.52 eV for the binary material and 1.61 eV for the ternary composite.

**Table 4:** Optical Energy Gap as Retrieved from Absorption Measurements

material	energy gap (eV)
BiOI	1.84
MOF	1.10
BiOI/MOF 30%	1.52
BiOI/MOF/F-BC 10%	1.61

### Fourier Transform Infrared Spectroscopy

**Figure 10:**
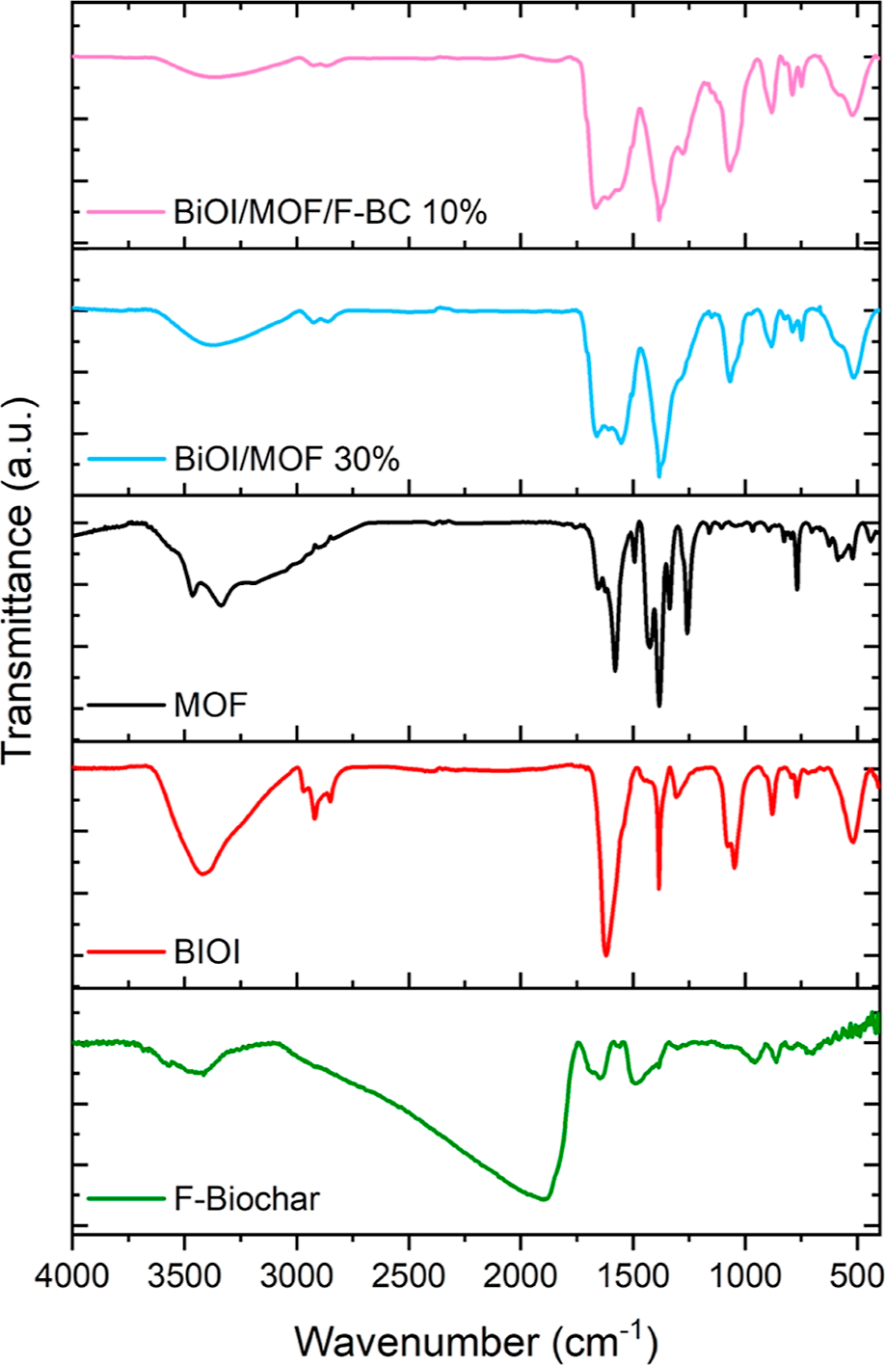
FTIR spectra of materials under investigation.

Fourier transform infrared (FTIR) spectroscopy was applied to investigate the formation of the composites by the comparative analysis of their vibrations with the signals pertaining to the bare materials (Figure 10).

The success of the acidic functionalization of the biochar was confirmed by the presence of a very intense and extremely broad peak centered at 1895 cm^–1^, ascribed to the C=O stretching of the carboxylic groups. Signals centered at 1650 and 1493 cm^–1^ were attributed to the asymmetric and symmetric stretching of the O–C=O groups, respectively.

BiOI infrared spectrum presents the peak typically ascribed to the Bi–O stretching vibration centered at 520.7 cm^–1^, together with an asymmetric sharp and rather intense signal at 1384.8 cm^–1^, due to the overlapping of symmetric and asymmetric stretching of the Bi–I bond.^7,31^ The analysis of the infrared spectrum of BiOI also revealed the presence of water molecules (H_2_O bending at 1620 cm^–1^ and O–H stretching at 3420 cm^–1^) and residuals of ethylene glycol from the synthesis (fingerprint triplet pertaining to the CH_2_ stretching at 2970, 2920, and 2850 cm^–1^ and doublet at 1078 and 1047 cm^–1^). Ethylene glycol residuals were also identified in the spectra of the binary and ternary composites.

The FTIR spectrum of MOF clearly showed the vibrations of amines (3462, 3336.7, 3184.3 cm^–1^ N–H stretch; 1575 cm^–1^ N–H bending; 1257.5 cm^–1^ C–N stretching; 769.6 cm^–1^ N–H wagging), as well as the peaks pertaining to the asymmetric and symmetric COO^–^ stretching (1658.7 and 1579.6, 1427 and 1393 cm^–1^, respectively). The breathing modes of the phenyl rings are also visible (623 cm^–1^), partly overlapping with the stretching of Fe–O (586 cm^–1^), which also presents a signal at 439 cm^–1^.

Several of these vibrations appear in the FTIR spectra of the binary and ternary composites as well; in both samples, an unresolved doublet is present at 520 and 578 cm^–1^, and this signal is the combination of the vibrations from the BiOI and the Fe–O bonds in the MOF. The loss of a clear identity of these vibrations from the bare materials suggests an interaction between the iron and the bismuth species in the composites, which would account for their combined action in photocatalysis.

Overlapping of the C–N stretching with the symmetric COO^–^ stretching results in an unresolved triplet spanning from 1253 to 1382 cm^–1^, while the asymmetric COO^–^ stretching is still well visible at higher wavenumbers (1551 to 1660 cm^–1^). Around 3370 cm^–1^, the combined stretching of O–H and N–H is present. In the ternary composite, a further multiplet is also present (around 1850 cm^–1^), ascribed to the C=O stretching of the carboxylic functions produced in the functionalized biochar.

Summarizing the above, FTIR analysis provided further proof of the formation of the desired binary and ternary composites, featuring a combined structure in which BiOI was coupled with the MOF and the biochar.

### Correlating Material Characteristics with Functional Performance

**Figure 11:**
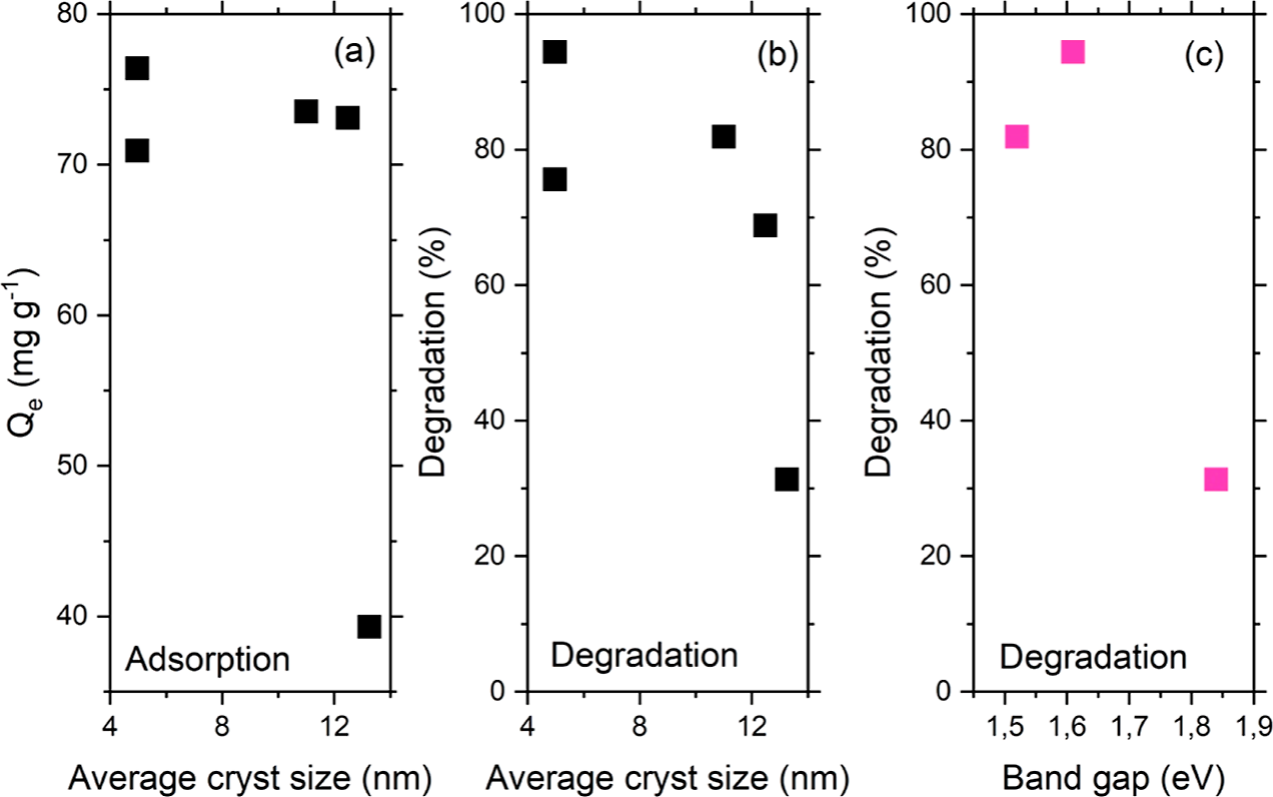
Dependence of adsorption at equilibrium and CIP photodegradation (at 180 min) as a function of (a,b) the average crystallite size and (c) the optical band gap.

We analyzed the performance of the materials in terms of their adsorption at the equilibrium and CIP degradation, focusing on BiOI as the main catalytic species, as a function of the crystallite sizes and the calculated optical band gap (Figure 11). We could observe an obvious dependence of both adsorption and CIP degradation on the crystallite sizes; the lower the latter is, the better the performance (Figure 11a,b).

This finding is a common claim in the literature analyzing the catalytic and photocatalytic performance of polycrystalline, nanostructured semiconductors; smaller crystallite sizes would enhance the number of catalytically active sites, thus spurring the degradation of targeted pollutants. No trend was found correlating the degradation of CIP under simulated solar light with the optical bandgap of the materials, and the modulation of this parameter does not affect the CIP removal from water. Eventually, this means that the modulation of light absorption plays a minor role in photocatalysis, surpassed by the structural features of materials. This finds confirmation also in the catalytic tests supported by the ternary composite and carried out in the presence of ROS scavengers; the photogenerated holes are the species playing the smallest role in degrading CIP.

### High-Performance Liquid Chromatography Coupled with Mass Spectrometry

We employed high-performance liquid chromatography–mass spectrometry (HPLC–MS) analysis to investigate the reaction mixtures and ascertain that the products generated in the CIP photodegradation are catalyzed by 10% BiOI/MOF/F-BC under different pH conditions. We then identified a pH-dependent degradation of the CIP (as demonstrated in Figure S7 in the Supporting Information), which has been previously documented in the literature, validating our research findings.^32−35^

The predominant photolytic pathway for CIP in acidic media involves oxidation of the piperazine ring and cleavage of the C–F bond.^32,34^ As a result of these reactions, a number of intermediates can be formed, including those with *m*/*z* values of 186, 205, and 217. These intermediates can undergo subsequent reactions and degradation pathways, as documented in the existing literature.^35,36^ The above masses were detected for the reaction mixture at pH 3 with BiOI/MOF/F-BC 10%, albeit with low intensity, indicating the possibility that the same mechanism is followed. The specific oxidation products formed during the degradation of piperazine-containing compounds may vary depending on the specific conditions and the presence of other reactive species. Moreover, when the reaction is carried out at pH 3, a compound featuring a parent ion with *m*/*z* of 332 is observed, indicating incomplete degradation of CIP.

A comparable degradation pathway was observed even at higher pH values (6.2, 9, and 11), resulting in the complete degradation of CIP into fragments with low molecular masses.

**Figure 12:**
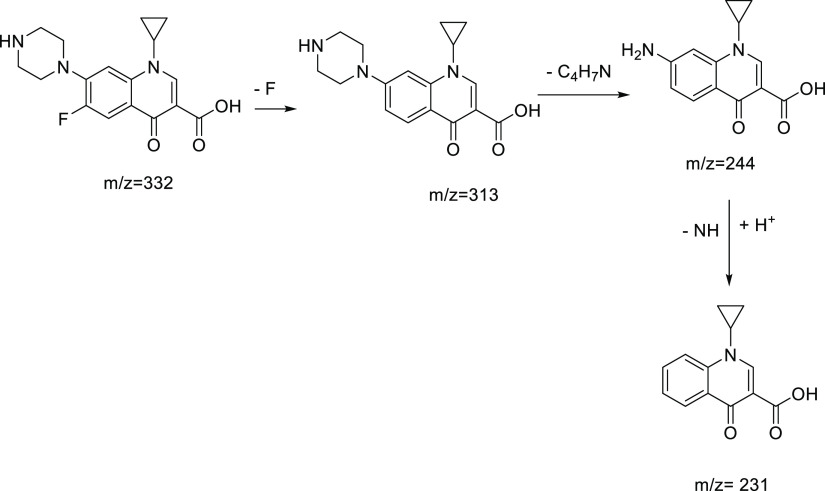
Proposed mechanism for CIP degradation catalyzed by BiOI/MOF/F-BC 10%.

Specifically, the presence of *m*/*z* = 231 was detected at pH = 6.2 and 9, while *m*/*z* = 245 was detected at pH = 11, indicating the involvement of the following degradation pathway (Figure 12).^36^

It is worth noting that some intermediates were not detected at different pH values, *e.g.*, *m*/*z* = 313 at pH 6.2 and 9; and *m*/*z* = 231 and 313 at pH = 11.

The degradation of CIP is favored in highly alkaline media. The presence of alkaline conditions promotes the formation of hydroxyl radicals through reactions with hydroxide ions and other reactive species. Hydroxyl radicals are known for their high reactivity and play a crucial role in initiating the degradation of CIP.

These results are consistent with the photocatalytic degradation finding, which indicates partial degradation of CIP at pH 3 and complete degradation at higher pH values (pH = 6.2–11). In addition, the results are also consistent with what was observed in the test carried out with scavenging agents, indicating the efficiency of superoxide anion radicals and hydroxyl radicals in CIP removal at higher pH values.

## Conclusions

A batch of binary and ternary compounds based on BiOI were synthesized, aimed at empowering this semiconductor photocatalyst with a MOF and a functionalized biochar. BiOI was thus combined with different amounts of NH_2_-MIL-101, and the best photocatalyst of this batch was used as the base material for the synthesis of a series of ternary hybrid composites with a COOH-functionalized biochar. All of the materials were then tested as adsorbers and photocatalysts for the removal of CIP from water.

The experimental outcomes showed that (i) the combination with MOF and F-BC drastically enhanced BiOI properties as both adsorber and photocatalyst; (ii) the fabricated composites are interesting adsorbers and photocatalysts for the removal of CIP from water; and (iii) there is a clear dependence between the adsorption at the equilibrium and the amount of degraded CIP upon simulated solar light irradiation on BiOI average crystallite size, which results in the main parameter determining the performance of the investigated materials. The role of NH_2_-MIL-101(Fe) is to enhance the adsorption capability of the materials, as observed through quantification of the adsorption at equilibrium; the induced proximity effect subsequently spurs CIP degradation under light excitation. The *in situ* addition of biochar, then, causes a significant decrease of BiOI crystallite sizes, which further promotes the catalytic action of the semiconductor. The combined action of these two chemicals on BiOI results in the empowerment of its functional performance.

The best photocatalyst of the investigated batch was tested at different pH conditions: these tests resulted in different amounts of CIP being removed from the original solution, as expected. However, the interesting outcome was that increasing the pH of the solution (compared to the pH induced by the spontaneous CIP dissociation in water) did not affect the amount of CIP degraded at prolonged reaction times (180 min).

The photocatalytic tests with scavenging agents showed that several ROS are involved in CIP degradation, which is again confirmed as a complex process with kinetics driven in different ways by different ROS. This further confirms that in real scenarios, the best strategy to remediate waters is the combination of different techniques.^37^

The present investigation shows that the fabrication of hybrid composites is a winning strategy to enhance the performance of BiOI as both an adsorber and a photocatalyst for CIP removal, indicating, however, that crystallite sizes command the overall process.

## Methods

### Material Synthesis

For the synthesis of materials, precursors from Sigma-Aldrich (ACS reagent, ≥98%) were used as-received, without any additional purification.

#### Synthesis of BiOI

Bi(NO_3_)_3_·5H_2_O (0.98 g) was dissolved in 15 mL of ethylene glycol (EG) under vigorous stirring for 30 min. In another beaker, KI (0.33 g) was dissolved in 15 mL of EG under stirring for 30 min. The obtained KI solution was added dropwise to the Bi(NO_3_)_3_·5H_2_O solution under continuous stirring for 30 min. The reaction mixture was then transferred to a stainless-steel autoclave lined with Teflon and heated at 130 °C for 18 h. The precipitate was washed three times with distilled water and ethanol and, subsequently, dried in an oven at 60 °C. For a detailed discussion of the synthesis protocol, the reader is referred to ref (24).

#### Synthesis of NH_2_-MIL-101(Fe)(MOF)

0.89 g of FeCl_3_·6H_2_O and 0.29 g of 2-aminoterephthalic acid (2-ATP) were separately dissolved in 20 mL of *N*,*N*-dimethylformamide, under continuous stirring for 20 min. The FeCl_3_·6H_2_O solution was added to the 2-ATP solution and kept under magnetic stirring for 2 h. The reaction mixture was then sealed in a Teflon-lined autoclave and heated in an oven for 40 h at 110 °C. The product was washed with ethanol and distilled water and eventually dried overnight in an oven at 80 °C in an ambient atmosphere.

#### Synthesis of Binary Composites (BiOI/MOF-10,20,30,50%)

0.98 g of Bi(NO_3_)_3_·H_2_O (0.98 g) was inserted into 15 mL of ethylene glycol and added to previously prepared MOF dispersions in 10 mL of the same solvent (appropriate amounts of MOF were used to reach a final composition of the binary composites, with the BiOI to MOF ratio as high as 10, 20, 30, and 50% w/w). Each reaction mixture was stirred for 2 h. Thereafter, KI (0.33 g) was dissolved in 15 mL of EG and added dropwise to the reaction mixture under stirring for 2 h. The final mixture was loaded into a Teflon-line autoclave and heated for 18 h in an oven at 130 °C. The product underwent a thorough cleaning process involving the use of ethanol and distilled water for washing. Subsequently, it was carefully dried overnight in an oven set at 80 °C, maintaining an ambient atmosphere.

#### Acidic Oxidation of Biochar

The biochar used in the present work was prepared according to a previously published method.^38^

The functionalization of biochar was accomplished as follows: HNO_3_-40% and H_2_SO_4_-60% were mixed together with the 1/3(v/v) ratio and added to 200 mL of water. 2.5 g of biochar was added to the above solution and kept under reflux at 70 °C for 6 h. The oxidized biochar was washed with distilled water for 2 h until the pH of the solution reached a neutral condition and dried in an oven at 80 °C overnight.

#### Synthesis of Ternary Composites (BiOI/MOF/F-BC-5,10,20%)

Functionalized biochar was dispersed in 10 mL of DMF and sonicated for 1 h. The amounts of F-BC were calculated to reach a final composition of the ternary composites with a BiOI/F-BC w/w ratio as high as 5, 10, and 20%, respectively. The ratio of BiOI/MOF was kept at 30% with respect to the weight amount of MOF to BiOI.

**Scheme 1:**
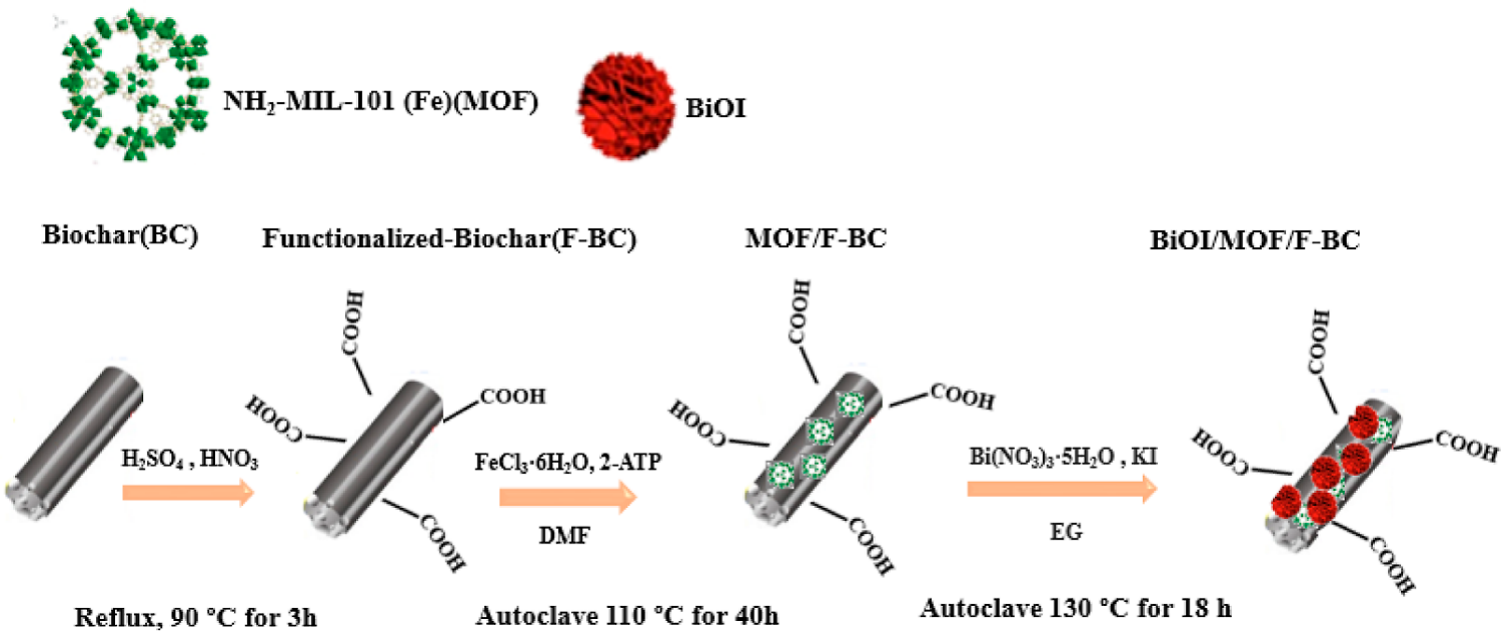
Synthesis of Ternary Composites BiOI/MOF/F-BC

FeCl_3_·6H_2_O (0.89 g) was added to the F-BC dispersions, which were stirred for 40 min, and 2-ATP (0.29 g) was then added. After 1 h of stirring, the reaction mixture was placed in an oven for 40 h at 110 °C. The obtained brown powder was dispersed in 15 mL of EG and sonicated for 20 min. 0.98 g of Bi(NO_3_)_3_·5H_2_O was then added to the dispersion, which was stirred for 1 h before adding dropwise (2 h) KI (0.33 g) dissolved in 15 mL of EG. The resulting precursor suspension was loaded into a Teflon-lined stainless-steel autoclave and kept at 130 °C for 18 h. Finally, the resulting material was washed and inserted into a vacuum oven overnight at 80 °C (the synthesis is shown in Scheme 1).

### Material Characterization

XRD analysis was performed in a PANalytical Empyrean instrument equipped with a PIXcel3D detector using Cu Kα radiation at 40 keV and 45 mA settings.

SEM was carried out in a JSM-IT300 scanning electron microscope equipped with an energy-dispersive X-ray detector (EDX).

FTIR spectra were recorded in a Bruker VERTEX 80v in the range 4000–400 cm^–1^ in transmittance mode.

Optical absorption spectroscopy was carried out in an Agilent Cary 5000 spectrophotometer.

Brunauer–Emmett–Teller (BET) of materials was investigated with a Micromeritics Gemini V 2390 apparatus (Micromeritics, Norcross GA, USA). The samples were degassed under vacuum (1 × 10^–4^ Pa) at 90 °C for 24 h before measurement.

Thermogravimetric analysis (TGA) was carried out through an STA 449C Jupiter (NETZSCH, Germany) instrument. Each sample (5–10 mg) was heated from 25 to 700 °C at a rate of 10 °C min^–1^ under an Ar flow.

#### Adsorption Tests

Ten mg of each adsorber species was added to 100 mL of CIP solution (10 ppm). The mixtures were kept in the dark and vigorously stirred for 120 min. Aliquots were withdrawn at fixed time intervals and analyzed with the help of spectrophotometry (Agilent Cary 5000 spectrophotometer) to determine the CIP disappearance by monitoring the evolution of the absorption peak centered at 276 nm.

#### Photocatalytic Tests

A LOT-QD solar simulator was used as the light source (AM 1.5G spectrum at 1 sun intensity, calibrated with a silicon reference cell). Ten mg of each catalyst was added to a 10 ppm CIP aqueous solution. Aliquots of the reaction mixtures were extracted at specific times and analyzed through spectrophotometry (Agilent Cary 5000 spectrophotometer) after centrifugation. CIP concentration was quantified by monitoring the evolution of the peak centered at 276 nm.

For the tests carried out under different pH conditions, HCl and NaOH were added to the solutions in appropriate amounts to reach pH 3, 9, and 11.

To analyze the role played by ROS in the photodegradation of CIP, 2 mM of various scavengers potassium iodide (KI, h^+^), *p*-benzoquinone (BQ, trapping the ^•^O_2_^–^), and isopropyl alcohol (IPA, ^•^OH) was added, following the same procedure as described in the photodegradation test.

#### High-Performance Liquid Chromatography–Mass Spectrometry

For HPLC–MS analysis, an Agilent 6130 quadrupole LC/MS instrument was employed with an electrospray ion source in positive ion mode. An adequate amount of the reaction mixture (2 μL) was injected into the HPLC column (C18, 50 × 3 mm with a 3 μm particle size and 100 Å pore size). In the mobile phase, a combination of water (solvent A) and acetonitrile (solvent B) (70:30) was used, both solvents containing 0.1% (v/v) formic acid.

## Supporting Information Available

Adsorption under dark *vs* F-BC content; fitting of adsorption process and related parameters; photodegradation *vs* MOF content; recyclability of BiOI/MOF/F-BC 10%; fitting parameters for the reaction courses; fitting of photodegradation supported by pure BiOI; structural parameters; XRD pattern of F-BC; and HPLC data (PDF)
