# Supplementary material for: Empowering Adsorption and Photocatalytic Degradation of Ciprofloxacin on BiOI Composites: A Material-by-Design Investigation
Source: ACS Omega. 2023 Nov 6;8(46):44044–56. doi: 10.1021/acsomega.3c06243 (PMC10666137; doi:10.1021/acsomega.3c06243)
Supplement: Supplementary file 1 — ao3c06243_si_001.pdf [file ao3c06243_si_001.pdf]

Empowering adsorption and photocatalytic degradation of ciprofloxacin

on BiOI composites:

A materials-by-design investigation

Sepideh G. Khasevani[a], Dariush Nikjoo[a], Cécile Chaxel[a], Kentaro Umeki[a], Shokat Sarmad[b],

Jyri-Pekka Mikkola[b, c], Isabella Concina[a*]

[a]Luleå University of Technology, Department of Engineering Sciences and Mathematics,

98187 Luleå, Sweden

[b]Wallenberg Wood Science Center, Department of Chemistry Technical Chemistry, Department of Chemistry, Chemical-Biological Centre, Umeå University, SE-90871 Umeå, Sweden

[c]Industrial Chemistry & Reaction Engineering, Johan Gadolin Process Chemistry Centre,

Åbo Akademi University, FI-20500 Åbo-Turku, Finland

* *Corresponding author: isabella.concina@ltu.se*

Supplementary Information

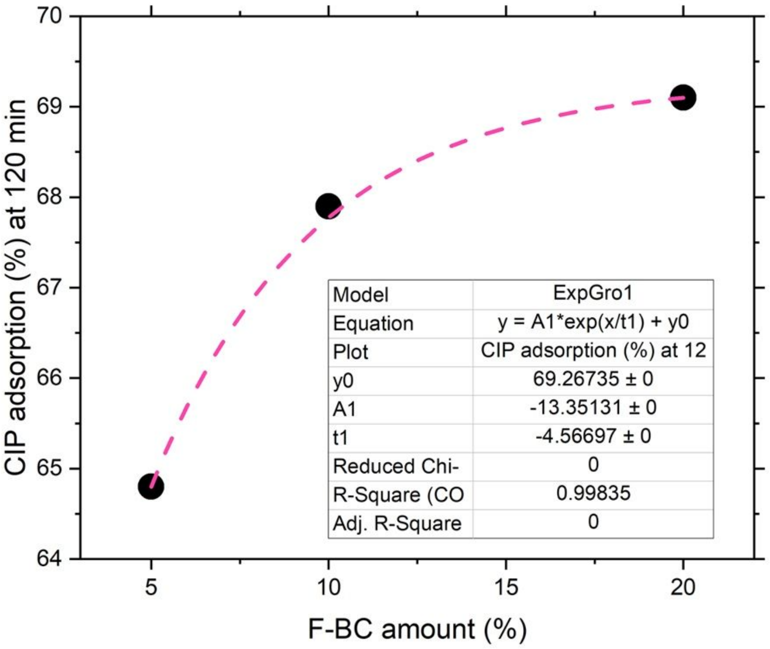

Figure S 1. CIP adsorption under dark vs. F-BC content for the ternary composites.

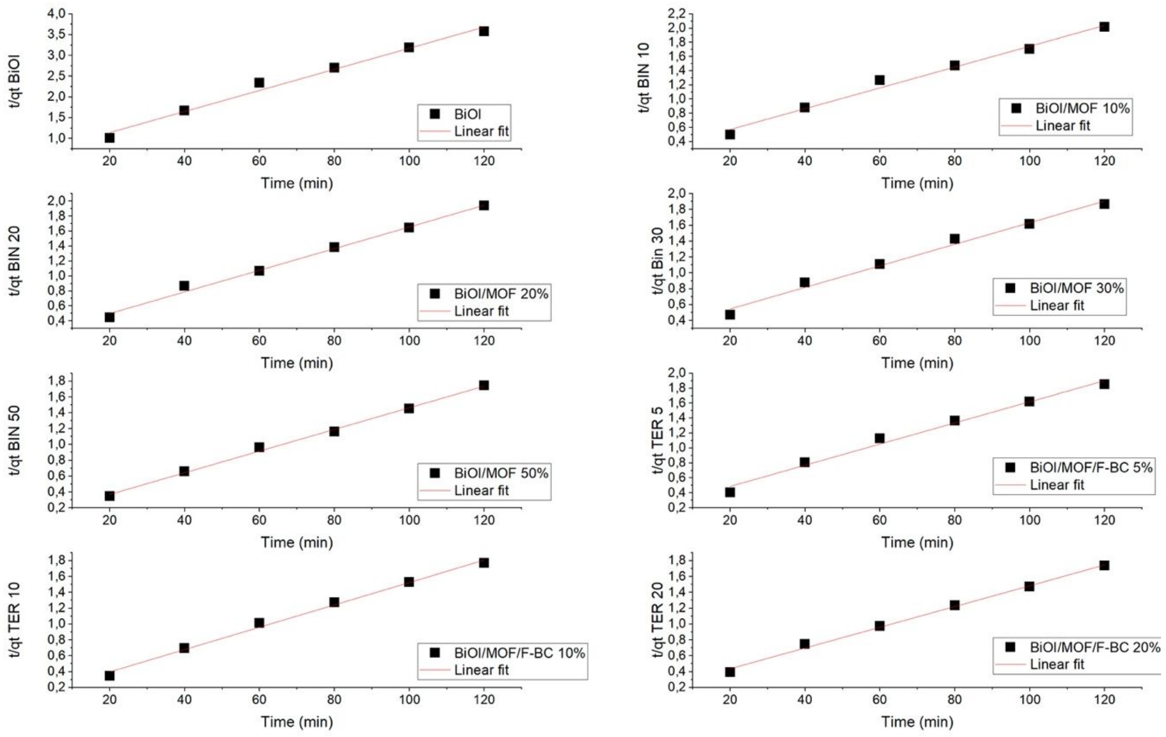

Figure S 2. Fitting according to a pseudo-second order model of the absorption process supported by the materials under investigation. Markers are experimental points, lines are fitting equations. Integrated equation: $q_t = \frac{q_e^2 k_2 t}{1 + q_e k_2 t}$.

Material	R^2	Slope (g mg^{-1})	Intercept (g mg^{-1} min)
BiOI	0.98599	0.02544 ± 0.00152	1.022
BiOI/MOF 10%	0.98690	0.01467 ± 0.06582	0.7698
BiOI/MOF 20%	0.99337	0.01446 ± 0.00059146	0.9936
BiOI/MOF 30%	0.98821	0.0136 ± 0.00074292	0.6739
BiOI/MOF 50%	0.99700	0.01368 ± 0.00037544	1.938
BiOI/MOF/F-BC 5%	0.98787	0.01415 ±0.00078370	0.9787
BiOI/MOF/F-BC 10%	0.99944	0.0141 ± 0.00053052	1.721
BiOI/MOF/F-BC 20%	0.99580	0.01309 ± 0.0004.2494	0.9751

Table S 1. Parameters of the fitting of the adsorption process for the materials under investigation.

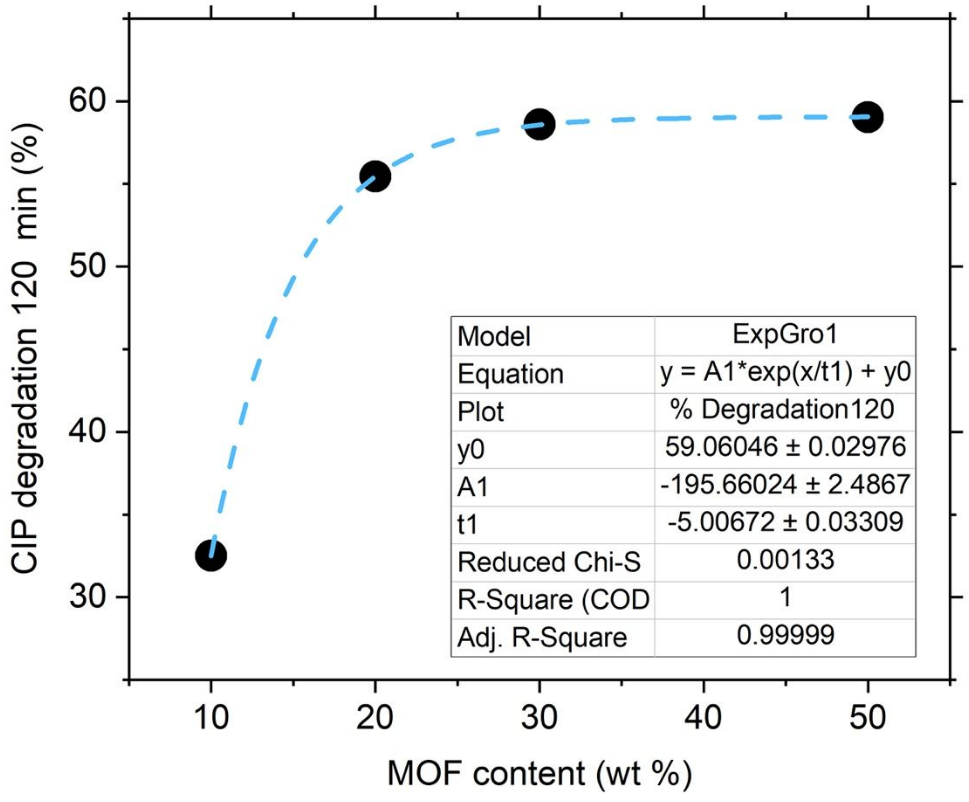

Figure S 3. CIP degradation under simulated solar light catalyzed by the binary composites vs. MOF content. Markers are experimental points; the dashed line is the exponential fit of the data.

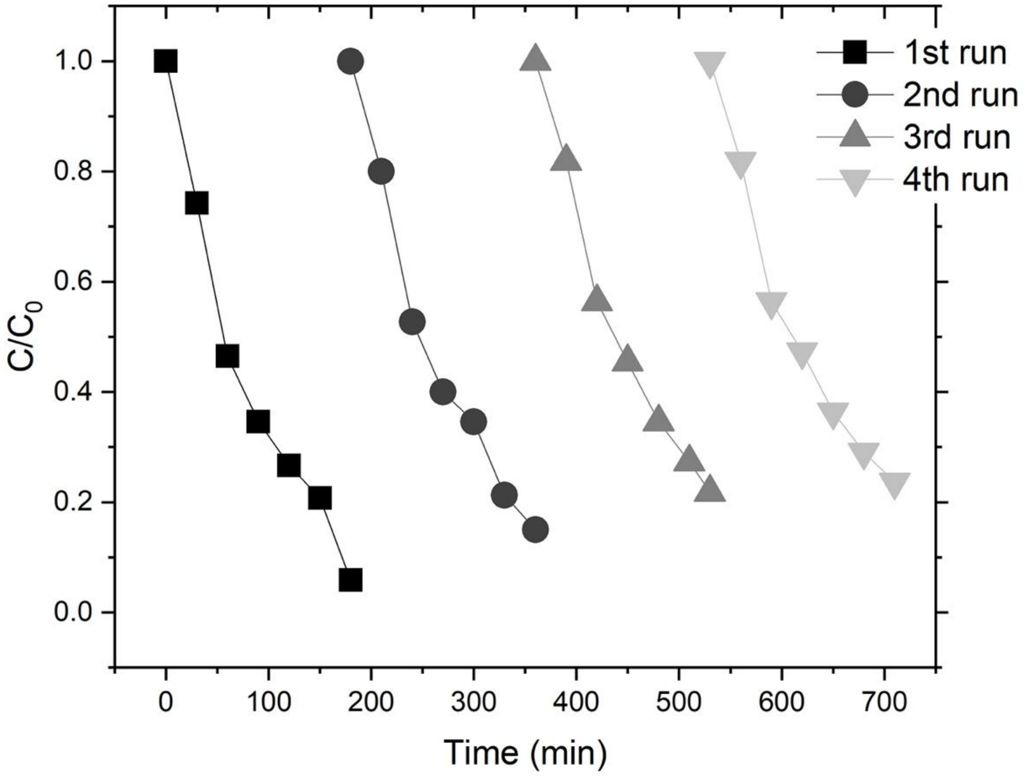

Figure S 4. Recyclability runs for the ternary composite BiOI/MOF/F-BC 10%.

**Material**	**$-\ln(C_t/C_0)$ *vs.* Time**	
	**Fitting Equations**	**R-Square**
MOF	Sigmoidal	$R^2 = 0.99947$
BiOI	Linear	$R^2 = 0.9896$
BiOI/MOF-30%	Linear	$R^2 = 0.99457$
BiOI/MOF/F-BC-10%	Linear AND exponential	$R^2 = 0.99295$<br>$R^2 = 0.99342$

Table S 2. Fitting parameters for the reaction course.

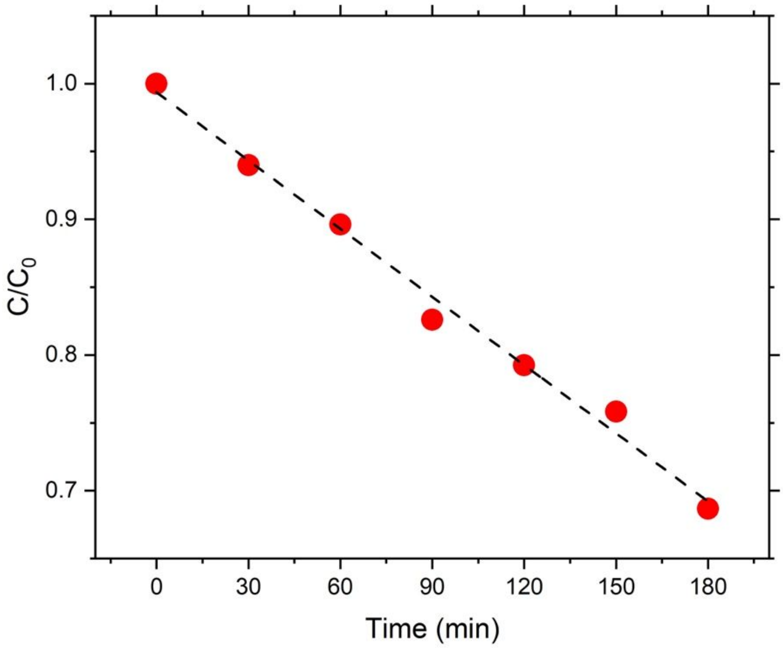

Figure S 5. Fitting of the reaction course for CIP degradation under simulated solar light catalyzed by pure BiOI.Markers are experimental point, line is the linear fitting (R^2=0.9912).

Material	2 Theta (°)	Reflection (hkl)	d-spacing (Å)
BiOI	8.70	001	10.1
	29.09	102	3.06
	31.77	110	2.81
	45.45	200	1.99
	55.09	212	1.66
BiOI/MOF 30%	29.35	102	3.03
	31.91	110	2.80
	45.73	200	1.98
	55.35	212	1.66
BiOI/MOF 50%	30.07	102	2.96
	32.09	110	2.78
	45.97	200	1.97
	55.94	212	1.64
BiOI/MOF/F-BC 10%	28.98	102	3.07
	31.81	110	2.81
	45.60	200	1.98
	54.88	212	1.67
BiOI/MOF/F-BC 20%	28.98	102	3.07
	31.81	110	2.81
	45.60	200	1.98
	54.88	212	1.67

Table S 3. XRD analysis, reporting the observed angles of reflection of different crystalline planes and the calculated interplanar distances (Bragg law was used, so that the interplanar distance is $d = \frac{n\lambda}{2 sin\,\theta}$).

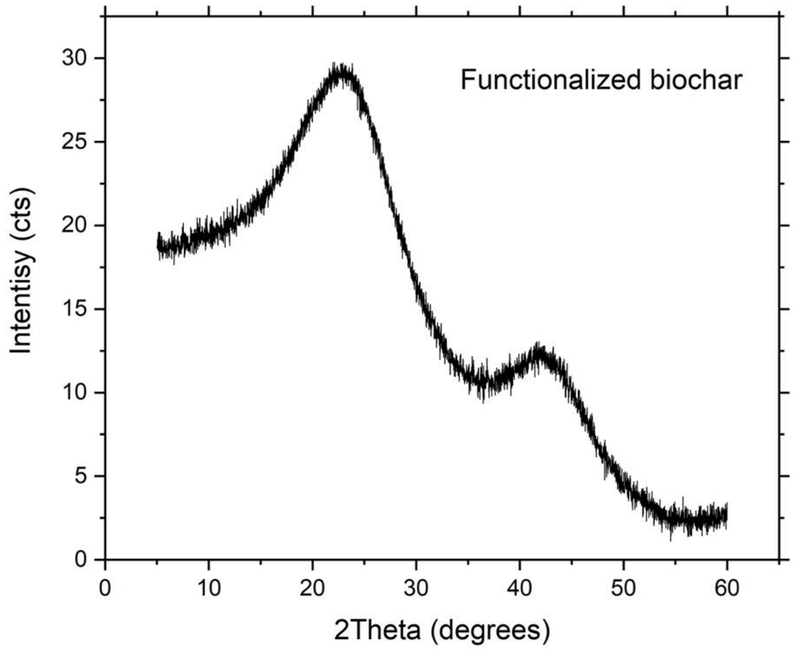

Figure S 6. XRD pattern of the oxidized biochar.

Figure S 7. UV spectrum along with the liquid chromatograms and mass-to-charge ratio spectra of the main photodegradation intermediates of ciprofloxacin generated in the photodegradation reaction catalyzed by the ternary composite (BiOI/MOF/F-BC 10%) at: (a) pH=3, (b) pH=6.2, (c) pH=9, and (d) pH=11.

a) pH=3

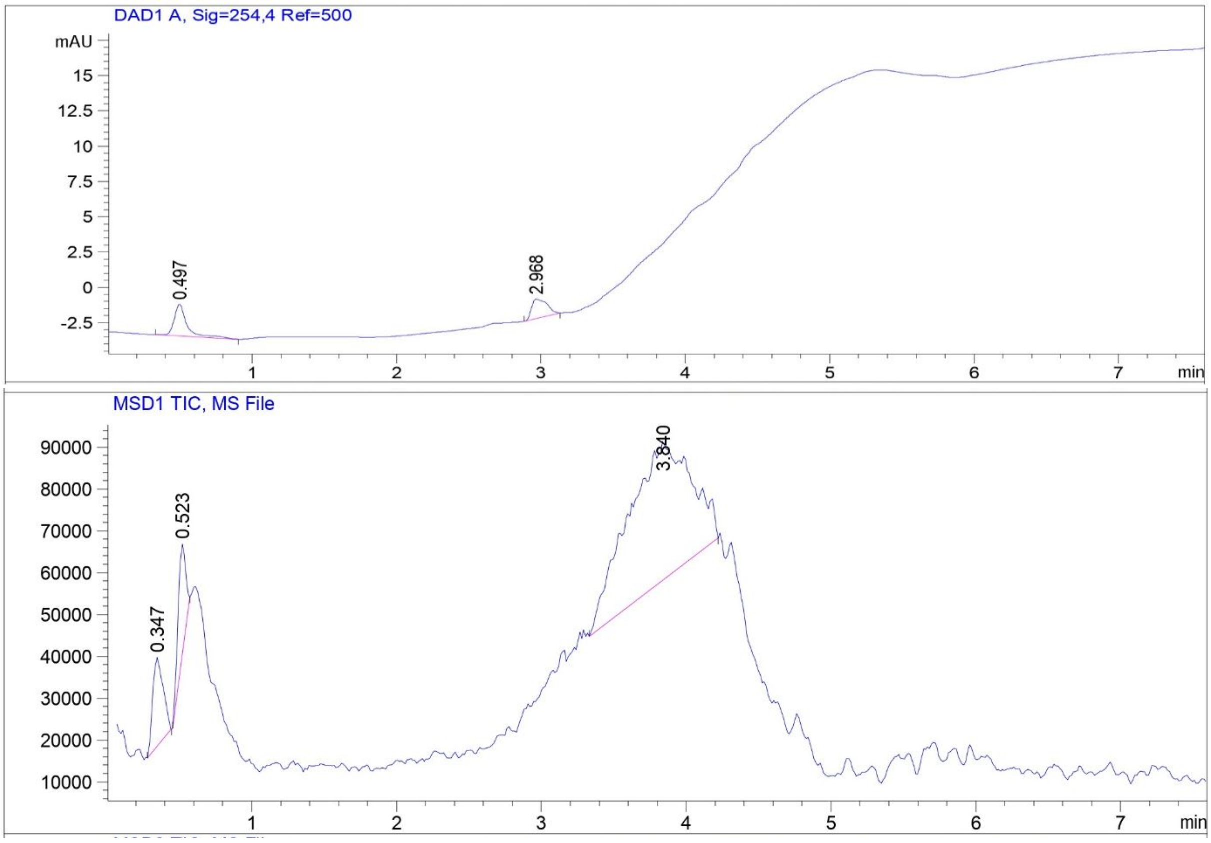

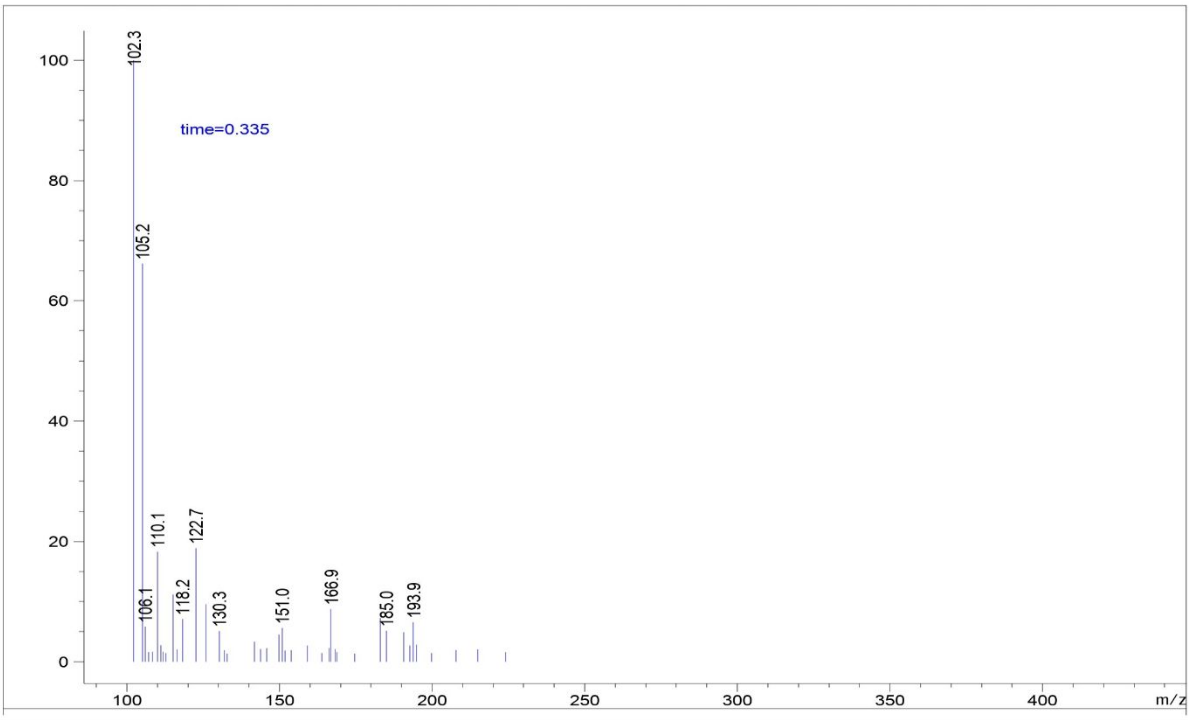

time=0.335
102.3
105.2
106.1
110.1
118.2
122.7
130.3
151.0
166.9
185.0
193.9
m/z

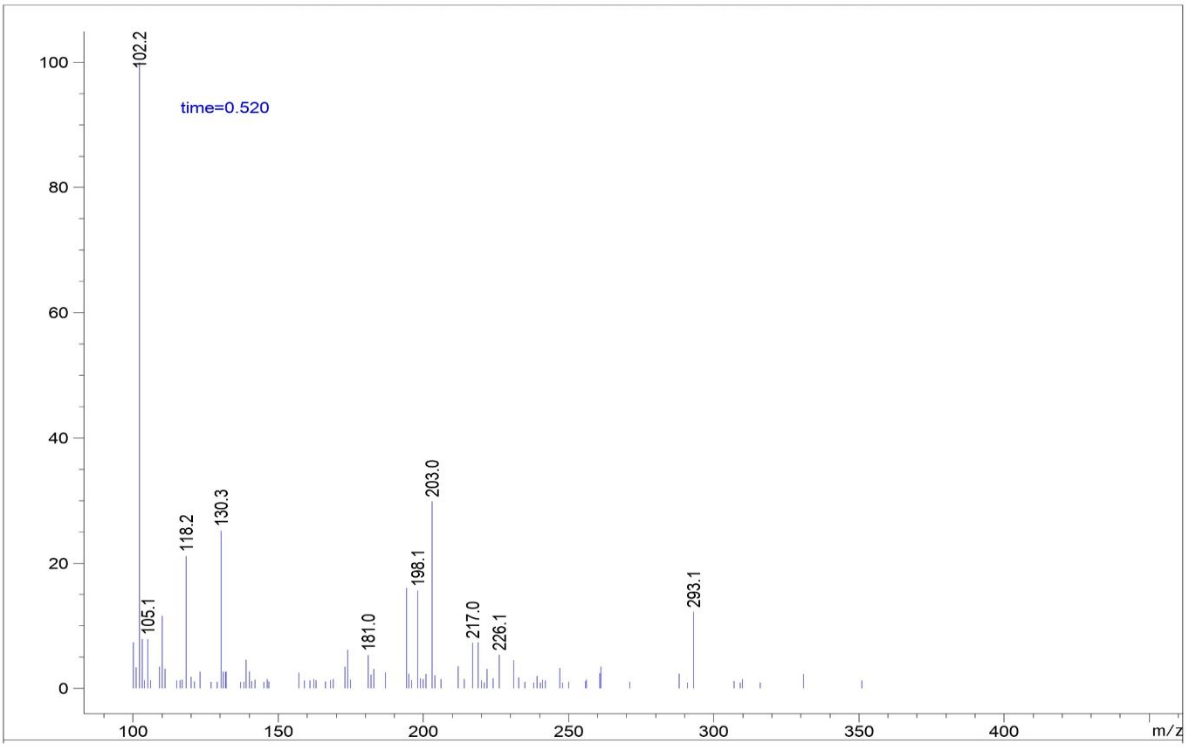

time=0.520
102.2
105.1
118.2
130.3
181.0
198.1
203.0
217.0
226.1
293.1
m/z

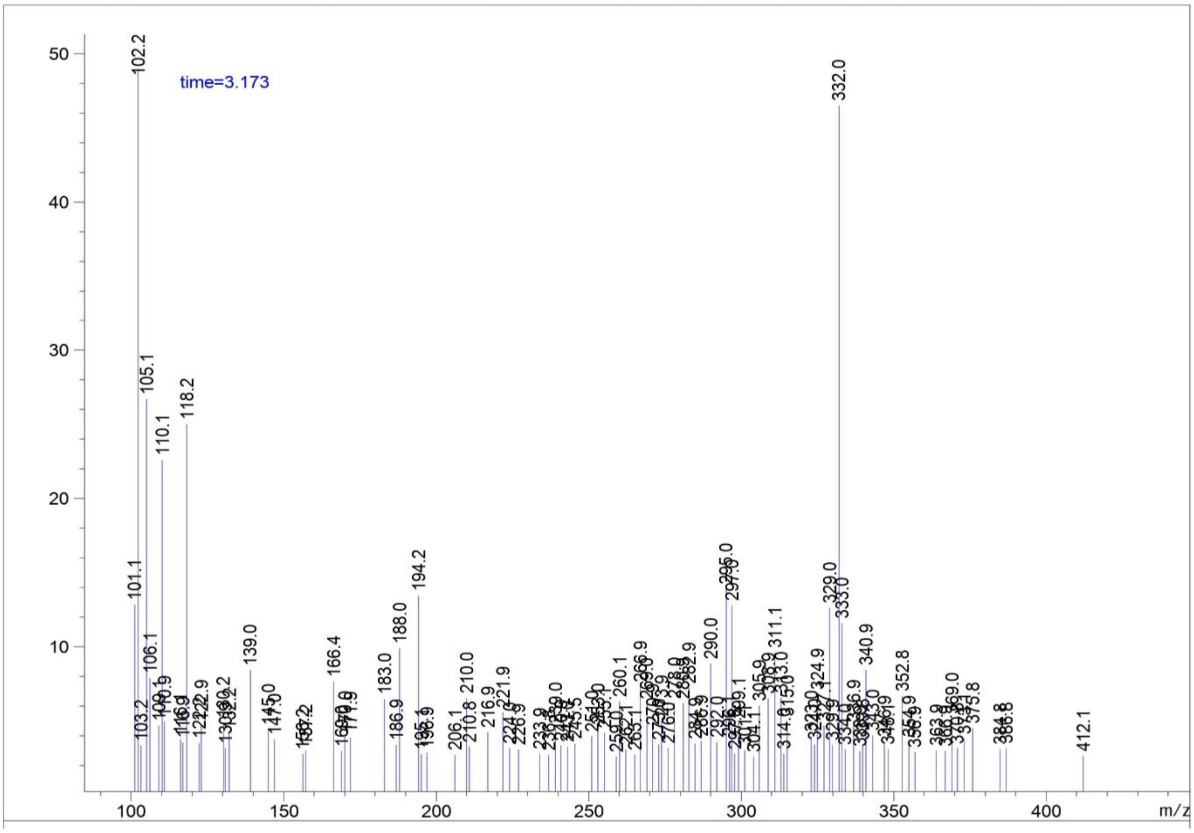

b) **pH=6.2**

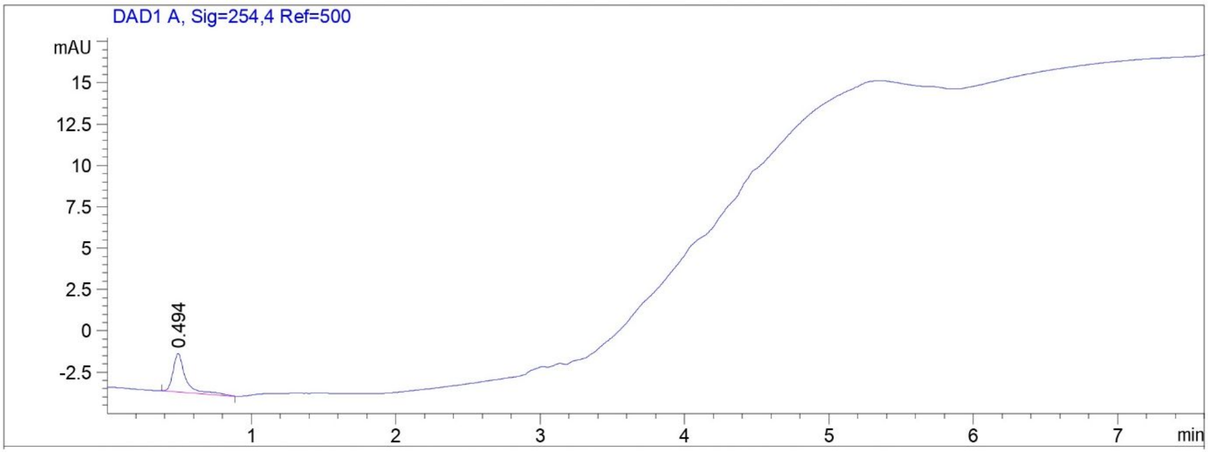

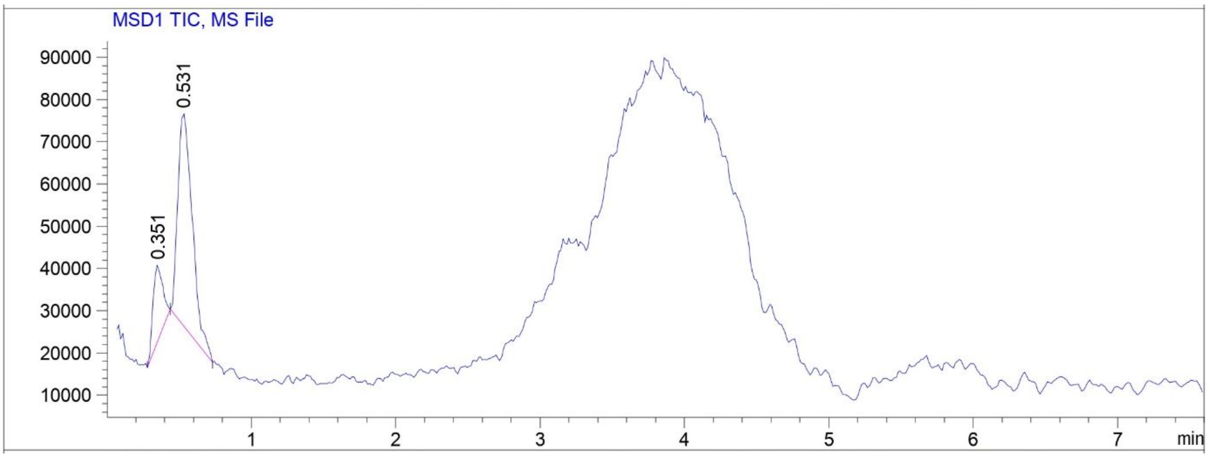
MSD1 TIC, MS File
90000
80000
70000
60000
50000
40000
30000
20000
10000
0.531
0.351
1
2
3
4
5
6
7
min

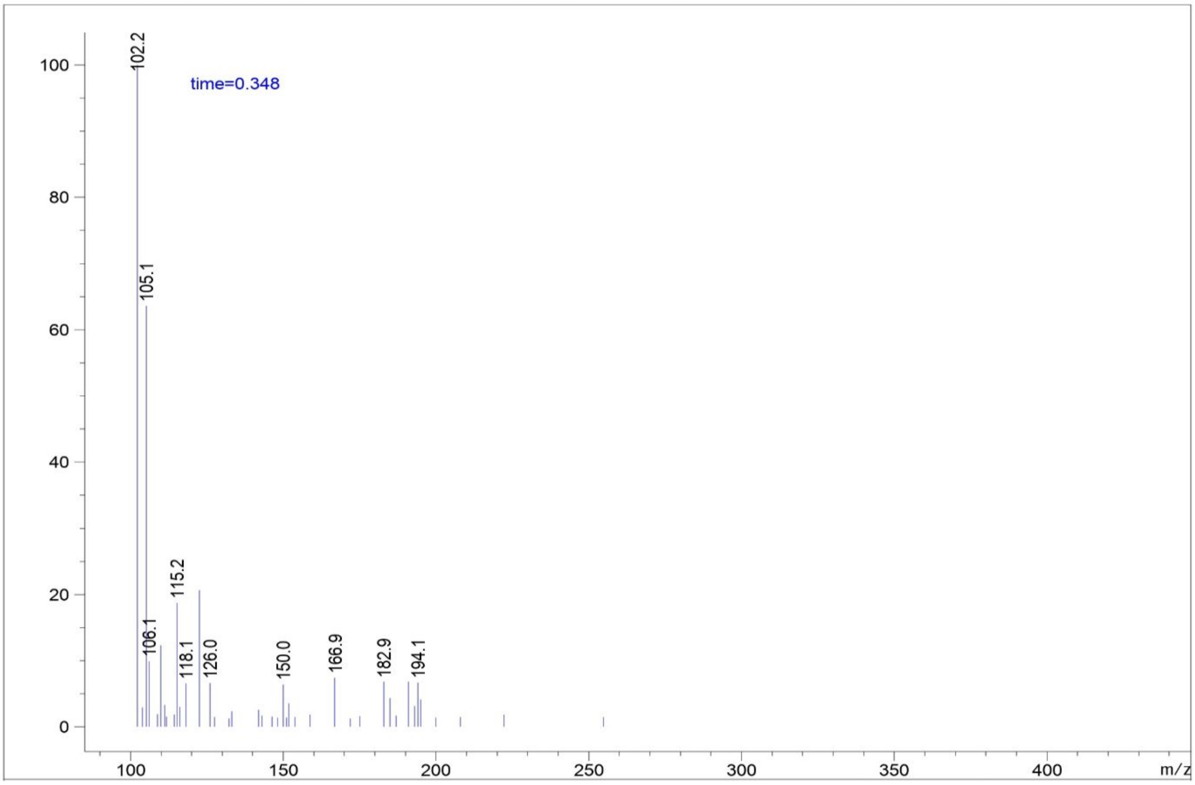
time=0.348
100
80
60
40
20
0
102.2
105.1
115.2
106.1
118.1
126.0
150.0
166.9
182.9
194.1
100
150
200
250
300
350
400
m/z

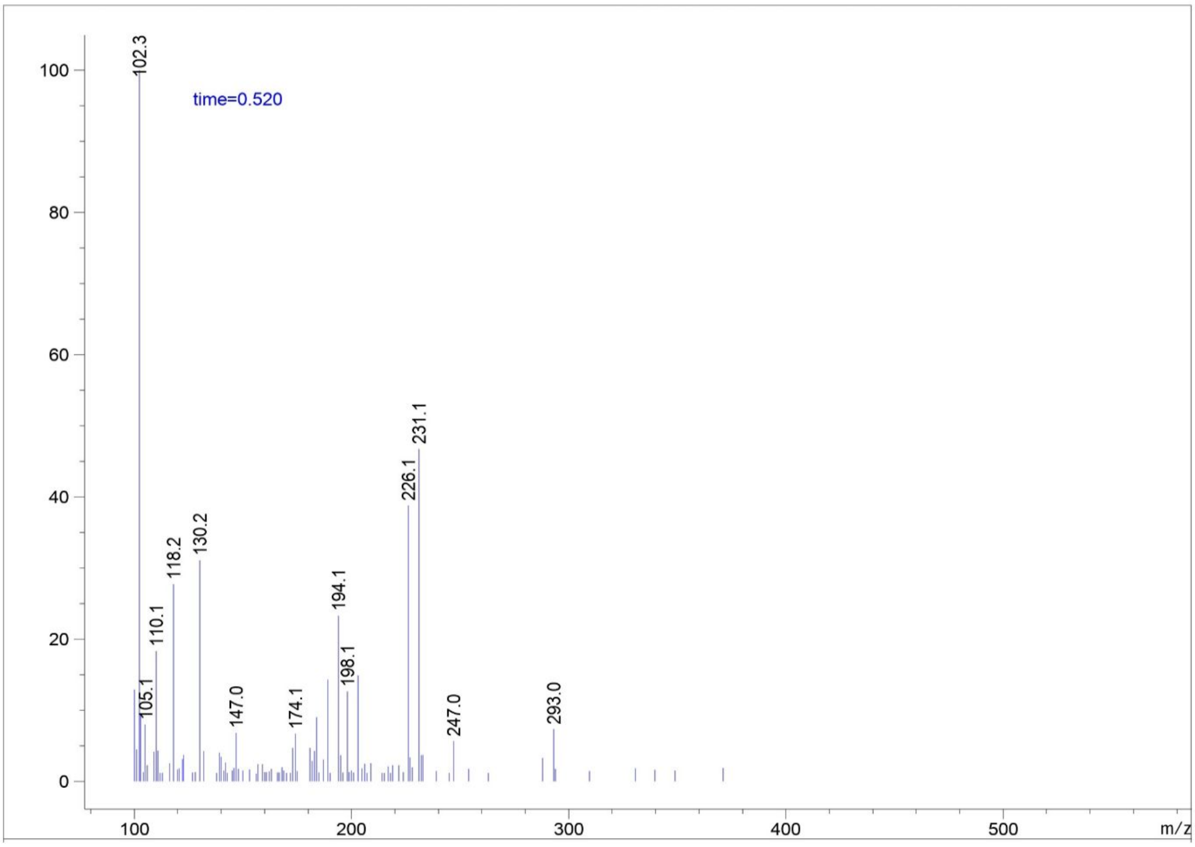
time=0.520
102.3
231.1
226.1
130.2
118.2
110.1
194.1
198.1
105.1
147.0
174.1
247.0
293.0
m/z

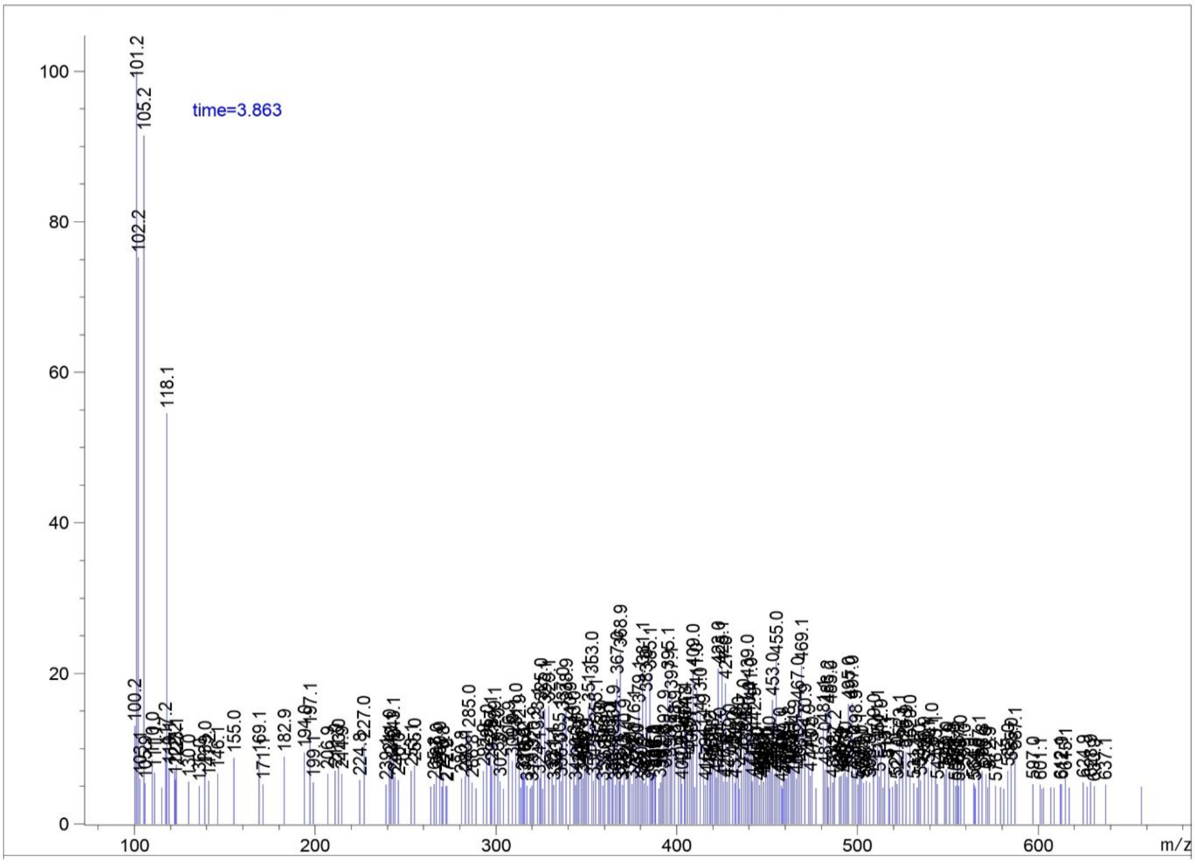
time=3.863
101.2
105.2
102.2
118.1
100.2
155.0
182.9
227.0
285.0
353.0
368.9
455.0
469.1
453.0
587.9
601.1
637.1
m/z

c) pH=9

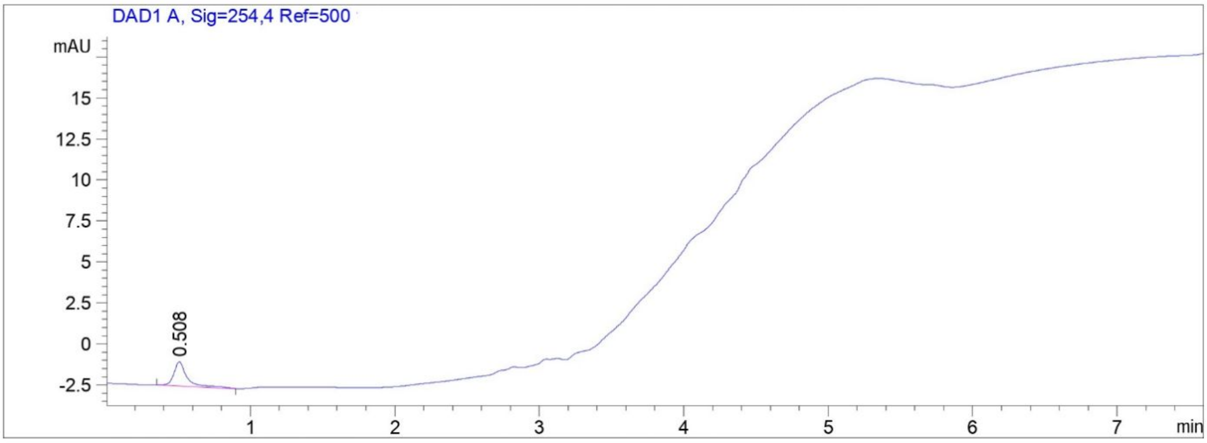
DAD1 A, Sig=254,4 Ref=500
mAU
15
12.5
10
7.5
5
2.5
0
-2.5
0.508
1
2
3
4
5
6
7
min

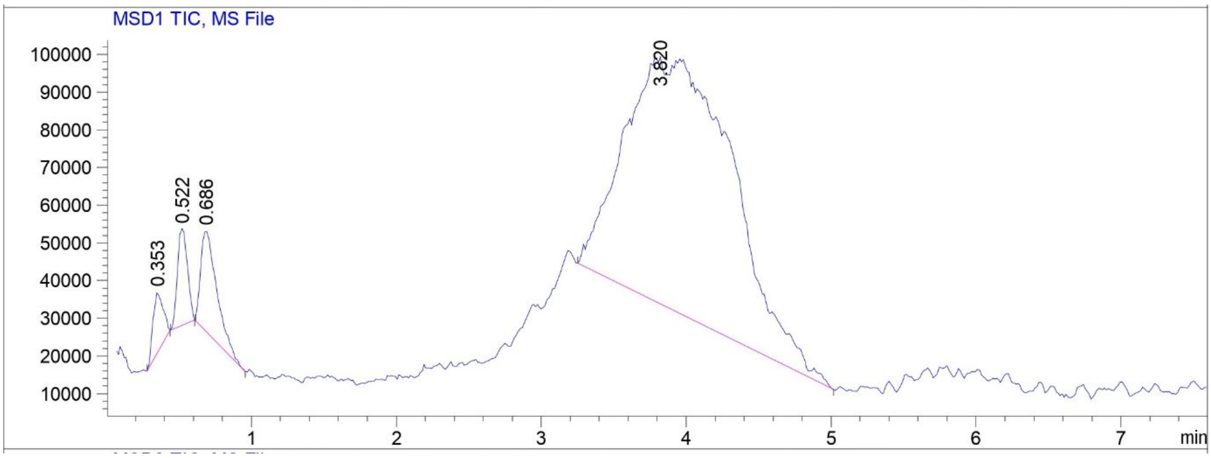
MSD1 TIC, MS File
100000
90000
80000
70000
60000
50000
40000
30000
20000
10000
0.353
0.522
0.686
3.820
1
2
3
4
5
6
7
min

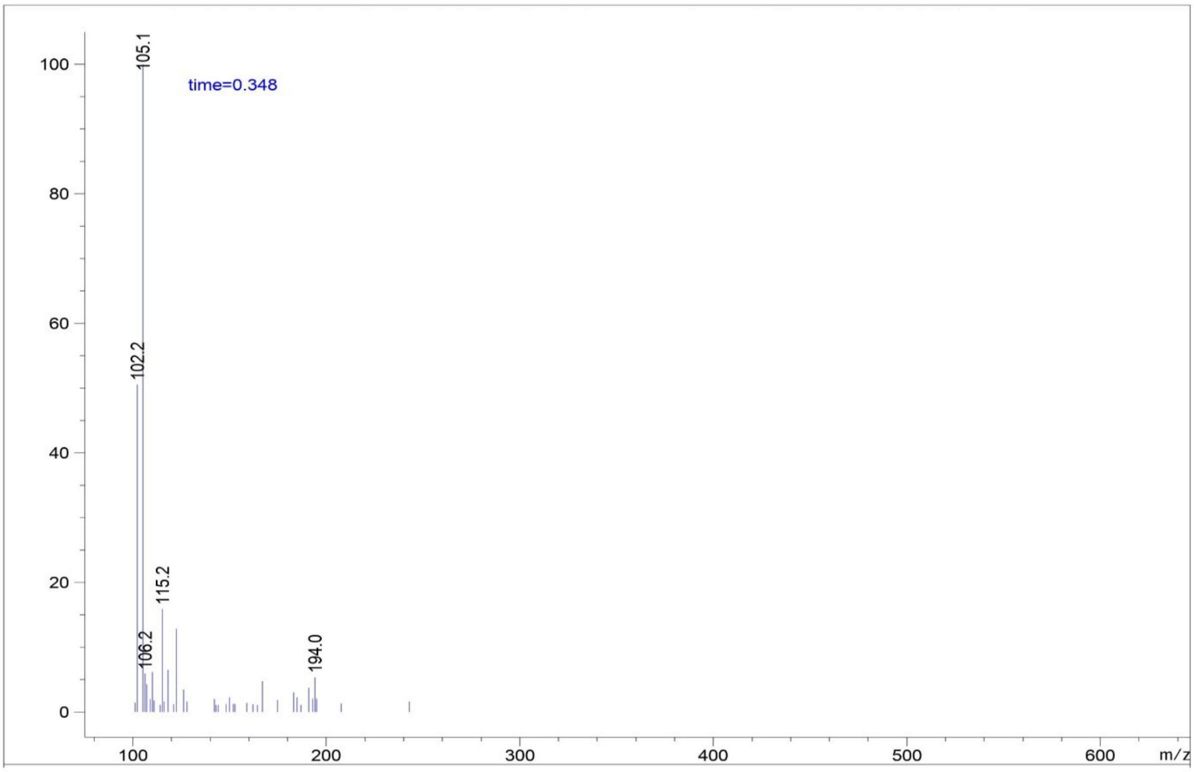

time=0.348
105.1
102.2
115.2
106.2
194.0
m/z

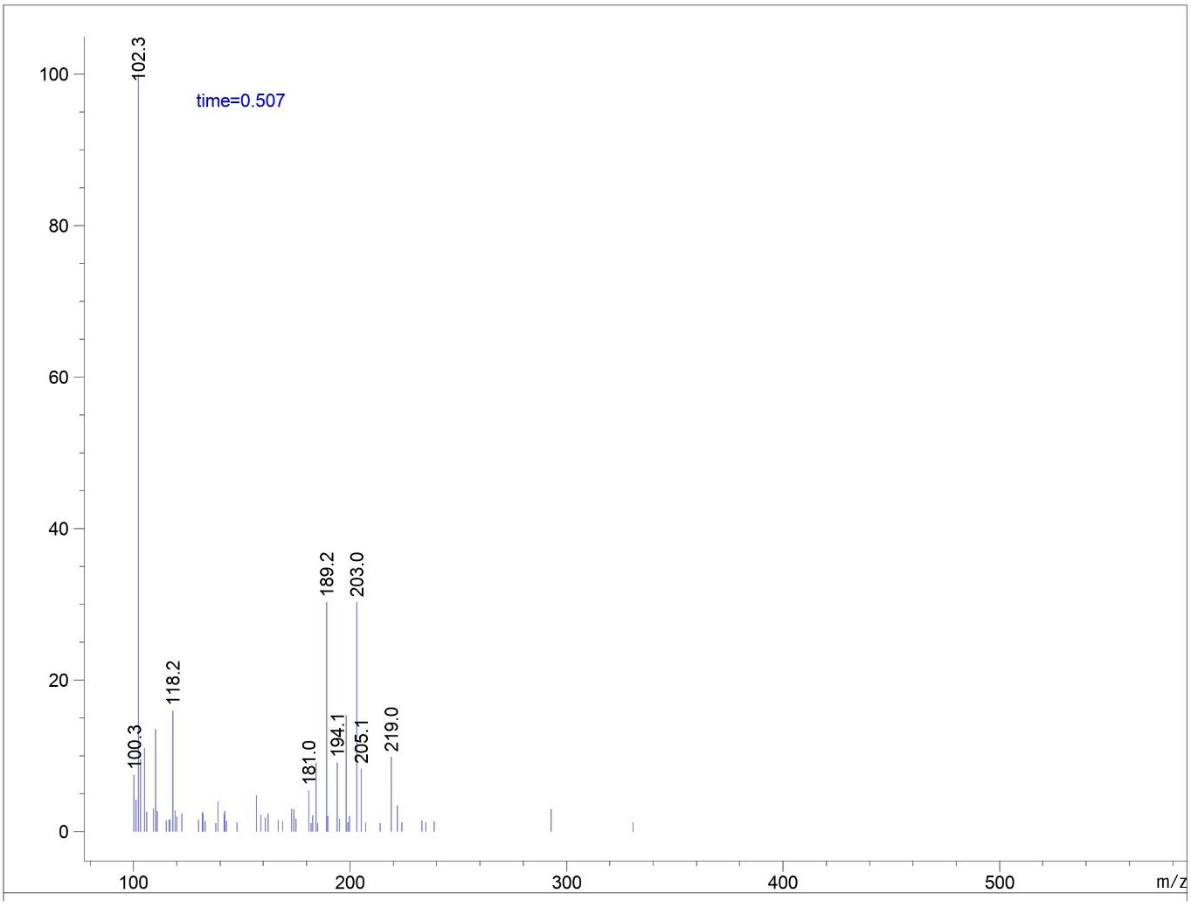

time=0.507
102.3
189.2
203.0
118.2
100.3
181.0
194.1
205.1
219.0
m/z

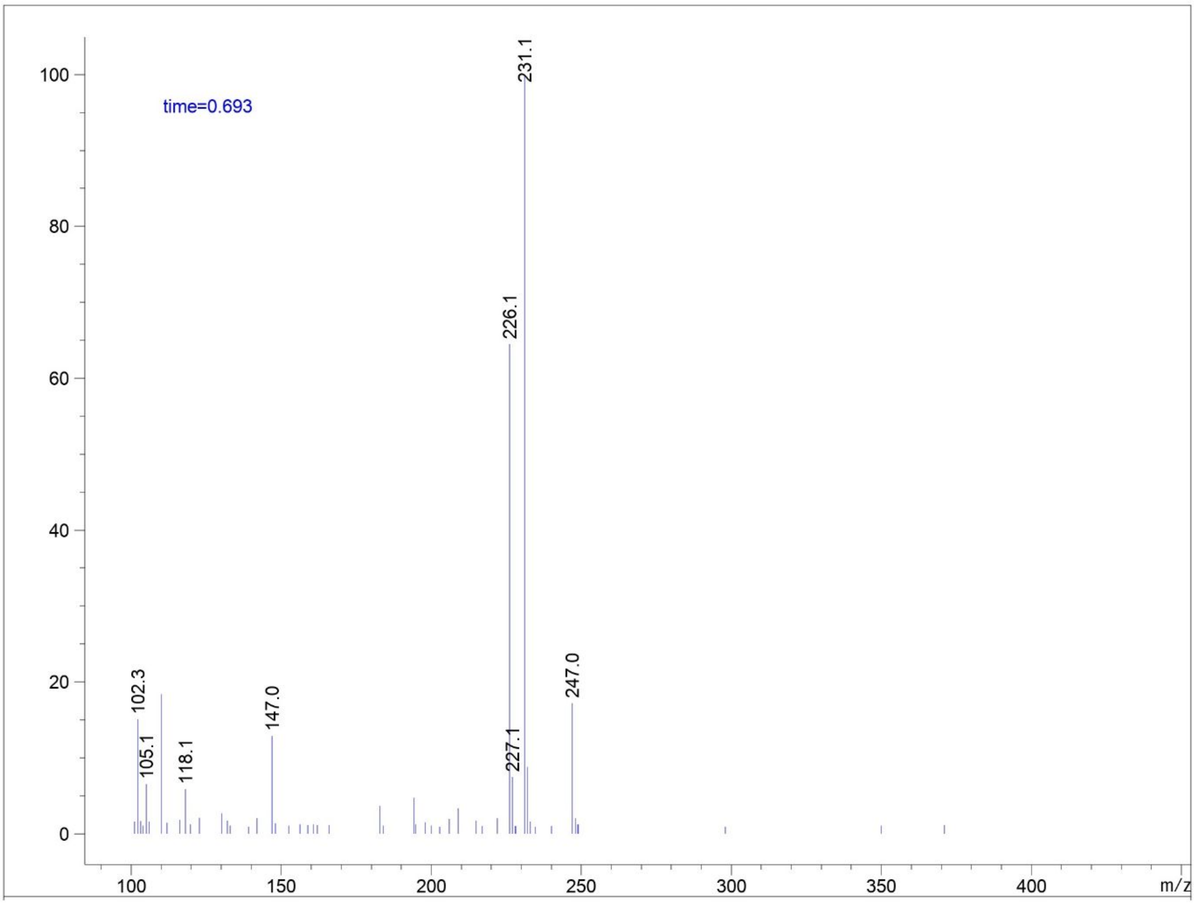

time=0.693
231.1
226.1
247.0
102.3
147.0
227.1
105.1
118.1
100
80
60
40
20
0
100
150
200
250
300
350
400
m/z

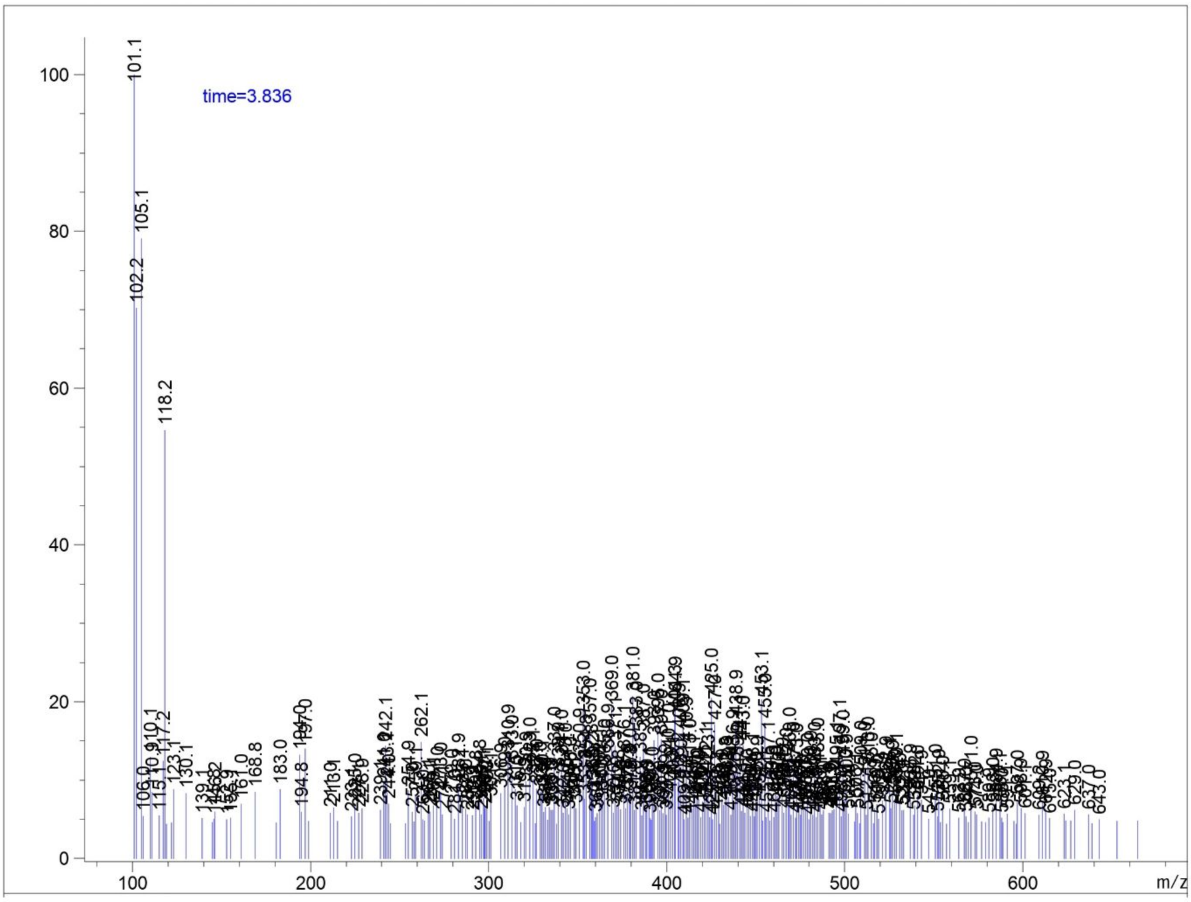
time=3.836
101.1
105.1
102.2
118.2
m/z

d) PH=11

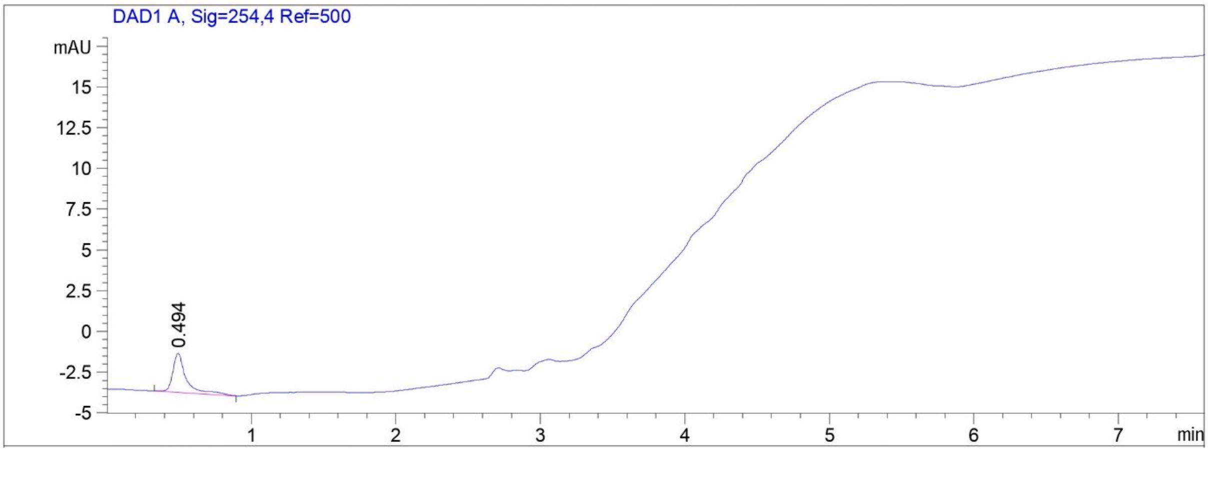
DAD1 A, Sig=254,4 Ref=500
mAU
0.494
min

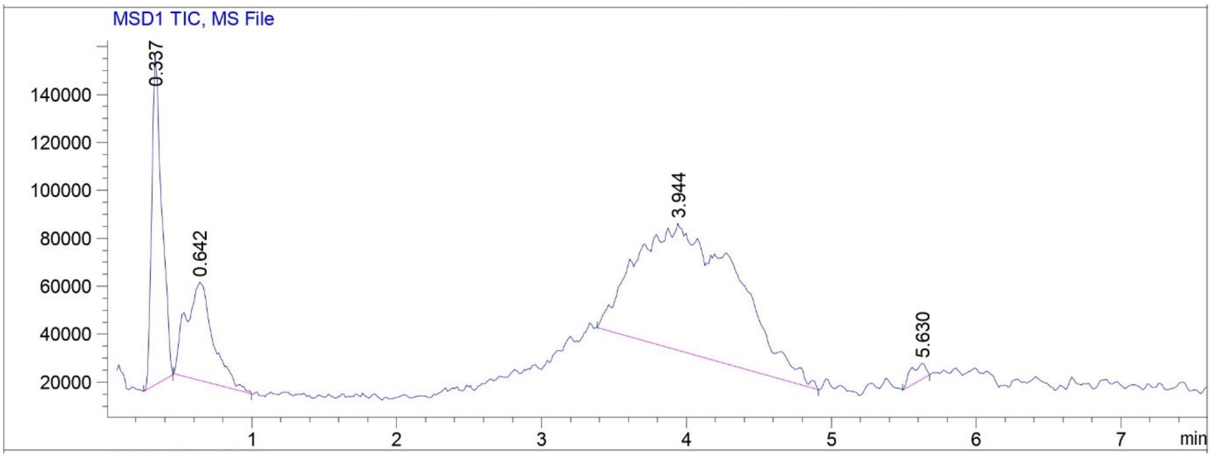
MSD1 TIC, MS File
0.337
0.642
3.944
5.630
140000
120000
100000
80000
60000
40000
20000
1
2
3
4
5
6
7
min

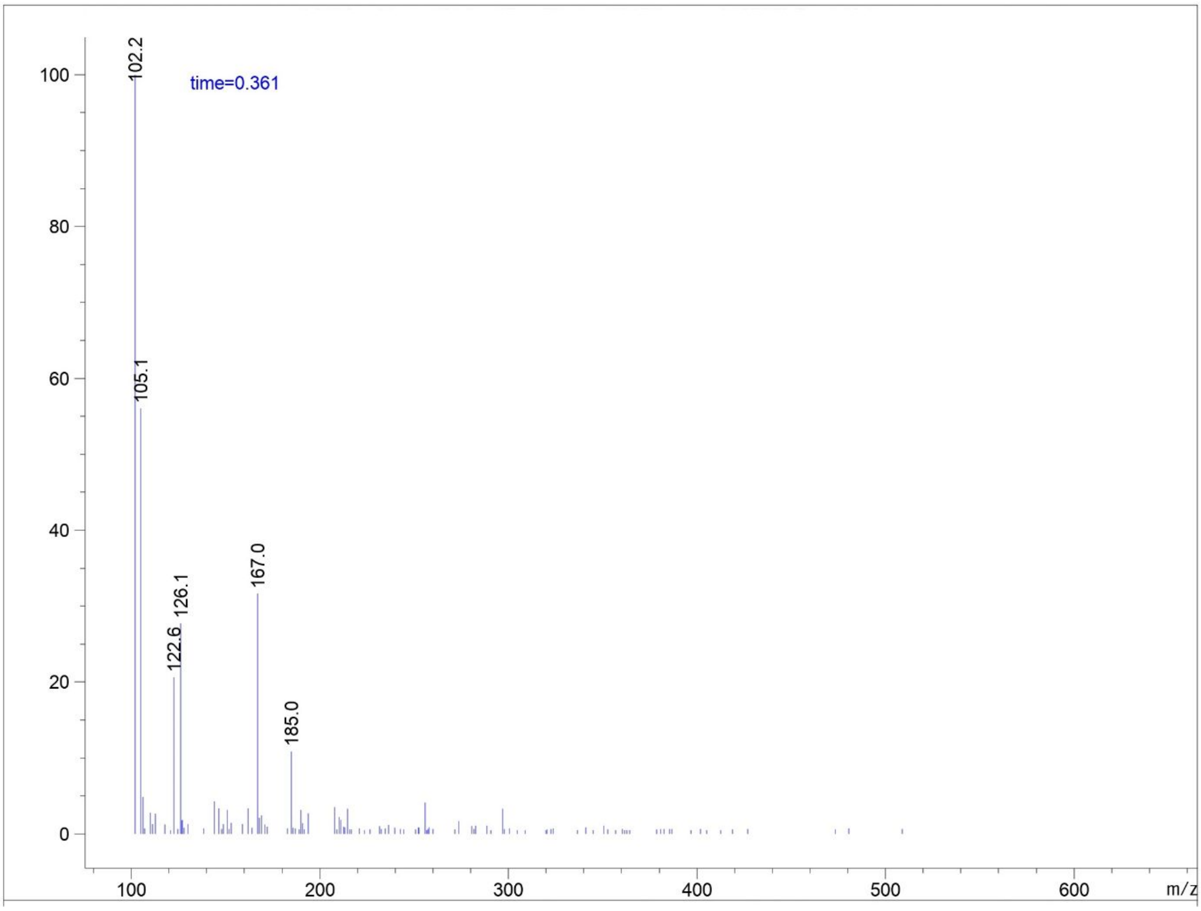
time=0.361
102.2
105.1
122.6
126.1
167.0
185.0
100
80
60
40
20
0
100
200
300
400
500
600
m/z

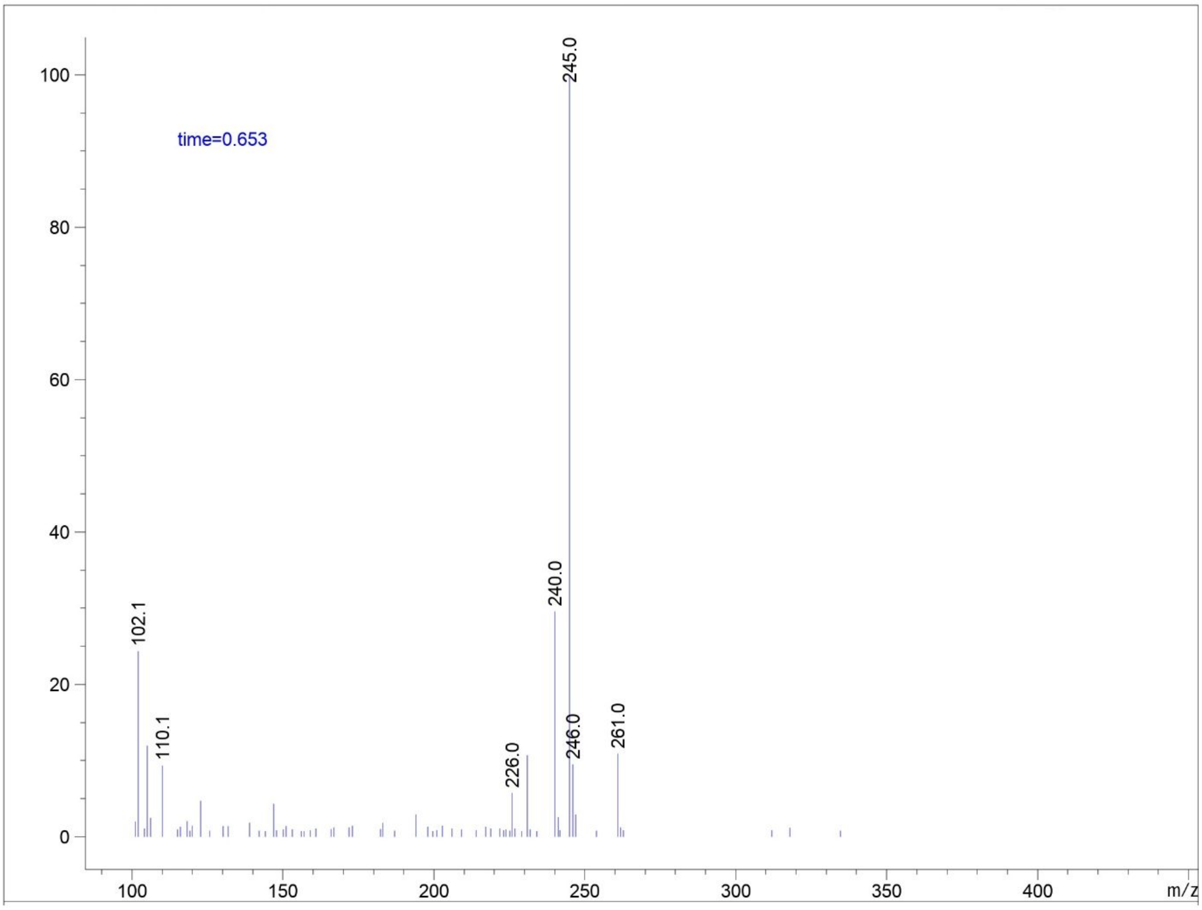
time=0.653
245.0
240.0
102.1
110.1
226.0
246.0
261.0
m/z
100
80
60
40
20
0
100
150
200
250
300
350
400

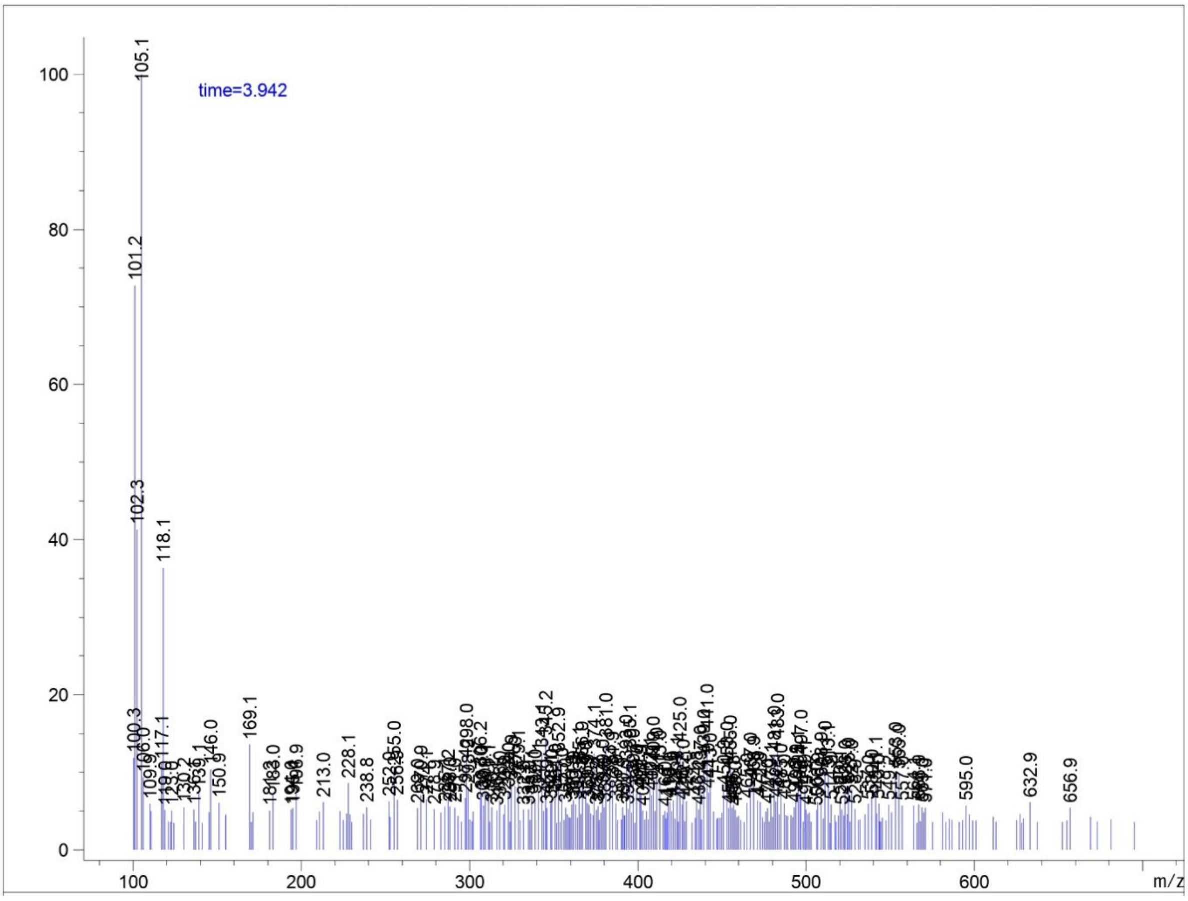
time=3.942
105.1
101.2
102.3
118.1
169.1
213.0
228.1
238.8
595.0
632.9
656.9
m/z
100
80
60
40
20
0
100
200
300
400
500
600
